# From Individual to Population: Circuit Organization of Pyramidal Tract and Intratelencephalic Neurons in Mouse Sensorimotor Cortex

**DOI:** 10.34133/research.0470

**Published:** 2024-10-07

**Authors:** Mei Yao, Ayizuohere Tudi, Tao Jiang, Xu An, Xueyan Jia, Anan Li, Z. Josh Huang, Hui Gong, Xiangning Li, Qingming Luo

**Affiliations:** ^1^ Britton Chance Center for Biomedical Photonics, Wuhan National Laboratory for Optoelectronics, MoE Key Laboratory for Biomedical Photonics, Huazhong University of Science and Technology, Wuhan, China.; ^2^ Research Unit of Multimodal Cross Scale Neural Signal Detection and Imaging, Chinese Academy of Medical Sciences, HUST-Suzhou Institute for Brainsmatics, JITRI, Suzhou, China.; ^3^ Department of Neurobiology, Duke University Medical Center, Durham, NC, USA.; ^4^ State Key Laboratory of Digital Medical Engineering, School of Biomedical Engineering, Hainan University, Haikou, China.; ^5^ Key Laboratory of Biomedical Engineering of Hainan Province, Hainan University, Haikou, China.

## Abstract

The sensorimotor cortex participates in diverse functions with different reciprocally connected subregions and projection-defined pyramidal neuron types therein, while the fundamental organizational logic of its circuit elements at the single-cell level is still largely unclear. Here, using mouse Cre driver lines and high-resolution whole-brain imaging to selectively trace the axons and dendrites of cortical pyramidal tract (PT) and intratelencephalic (IT) neurons, we reconstructed the complete morphology of 1,023 pyramidal neurons and generated a projectome of 6 subregions within the sensorimotor cortex. Our morphological data revealed substantial hierarchical and layer differences in the axonal innervation patterns of pyramidal neurons. We found that neurons located in the medial motor cortex had more diverse projection patterns than those in the lateral motor and sensory cortices. The morphological characteristics of IT neurons in layer 5 were more complex than those in layer 2/3. Furthermore, the soma location and morphological characteristics of individual neurons exhibited topographic correspondence. Different subregions and layers were composed of different proportions of projection subtypes that innervate downstream areas differentially. While the axonal terminals of PT neuronal population in each cortical subregion were distributed in specific subdomains of the superior colliculus (SC) and zona incerta (ZI), single neurons selectively innervated a combination of these projection targets. Overall, our data provide a comprehensive list of projection types of pyramidal neurons in the sensorimotor cortex and begin to unveil the organizational principle of these projection types in different subregions and layers.

## Introduction

The cerebral cortex has functional divisions, such as areas related to functions of movement, hearing, and vision. Motor actions can be induced by activating specific areas of the cortex, and the forelimb and hindlimb movement-related areas are responsible for the corresponding behavioral learning, planning, and execution [1,2]. The sensorimotor cortex is composed of multiple functional subregions with different behavioral consequences [3–8] and connectivity patterns [9], including the anterior–medial motor region (MOs^AMM^) and posterior–medial motor region (MOs^PMM^) of the secondary motor area, the intermediate–medial motor region (MOp^IMM^) and anterior–lateral motor region (MOp^ALM^) of the primary motor area, as well as the upper limb area (SSp-ul) and lower limb area (SSp-ll) of the primary somatosensory cortex. Importantly, the control of behavior is usually not limited to a single area, and a single area may be involved in multiple behaviors. For example, MOs^AMM^ participates in the selection of licking targets, motor planning, movement control, and other behaviors [5,6].

Each of these 6 regions contains different projection types of pyramidal neurons, including the pyramidal tract (PT) and intratelencephalic (IT) types with great differences in gene expression, morphology, electrophysiological property, and function [10,11]. PT and IT neurons form rich circuits connecting different target areas, including the different subregions within the sensorimotor cortex, the thalamus, brainstem areas, and the spinal cord [11–13]. These complex connections involve different neuronal types and highly specialized single-neuron activity. Individual neurons can be involved in multiple behaviors such that the activity of a single neuron may not predict overall behavior accurately [14]. The activity of neuronal populations is correlated with behaviors and often can be used to predict the overall behavior [15]. However, the brain circuit mechanism underlying the difference between population coding and single-neuron coding is not yet clear.

Previous work indicated that individual cortical neuron is active in multiple motor behaviors instead of in a specific single-motor behavior such as a flexion–extension finger movement [15,16]. Additionally, the spatial locations of neurons that are active in different motor behaviors overlap extensively in the sensorimotor cortex. These suggest that the control of bodily movement likely utilizes a distributed population of neurons [15]. Furthermore, stable behaviors are generated by stable single-neuron activity and neural dynamics of single neurons are highly stable [14]. Therefore, dissecting whole-brain connectivity at the level of individual neurons is crucial for delineating projection types of pyramidal neurons and for understanding the principle of signal transmission between brain regions. A series of studies on the sensorimotor cortex has found that there are many aspects of neuronal heterogeneity, including spatial location, morphological characteristics, gene expression, electrophysiological properties, and projection patterns [10,11,17–19]. However, there are still gaps in the research on the localization and connection patterns of different subregions and neuronal types, including the projection heterogeneity of the same transcriptomic type in different subregions or layers, which requires more and more detailed data analysis.

In this study, by using Cre driver mouse lines and low-dose tamoxifen induction, we sparsely labeled pyramidal neurons in the sensorimotor cortex and traced their axons across the entire brain with the fluorescence micro-optical sectioning tomography (fMOST) system to obtain single cell-level morphological datasets. We acquired the complete morphology of 1,023 pyramidal neurons in the sensorimotor cortex via single-cell reconstruction and performed diverse morphological characterizations of these pyramidal neurons labeled in different subregions. Our results reveal that different regions or layers are composed of IT and PT neurons with diverse projection patterns, thus forming a complex network of neural connections.

## Materials and Methods

The morphological datasets of pyramidal neurons were acquired with neurotropic virus tracing and fMOST, including resin embedding, whole-brain imaging, reconstruction, and visualization. Details about the methods are available in the Supplementary Materials.

## Results

### Complete morphology of individual pyramidal neurons in 6 subregions

To investigate the circuit structure of different types of pyramidal neurons, we used the *Fezf2-2A-CreER* and *PlxnD1-2A-CreER* transgenic driver mice [20–22], which offer genetic handles to specifically access PT and IT neurons, respectively [10,11,21]. To validate the expression pattern, we crossed the 2 driver mouse lines with the *Ai3* reporter mouse [expressing enhanced yellow fluorescent protein (eYFP) under the control of Cre]. As expected, we found that *Fezf2^+^* neurons are located in layers 5 and 6, while the *PlxnD1^+^* neurons are concentrated in layers 2/3 and 5a (Fig. S1A and B).

**Fig. 1.**
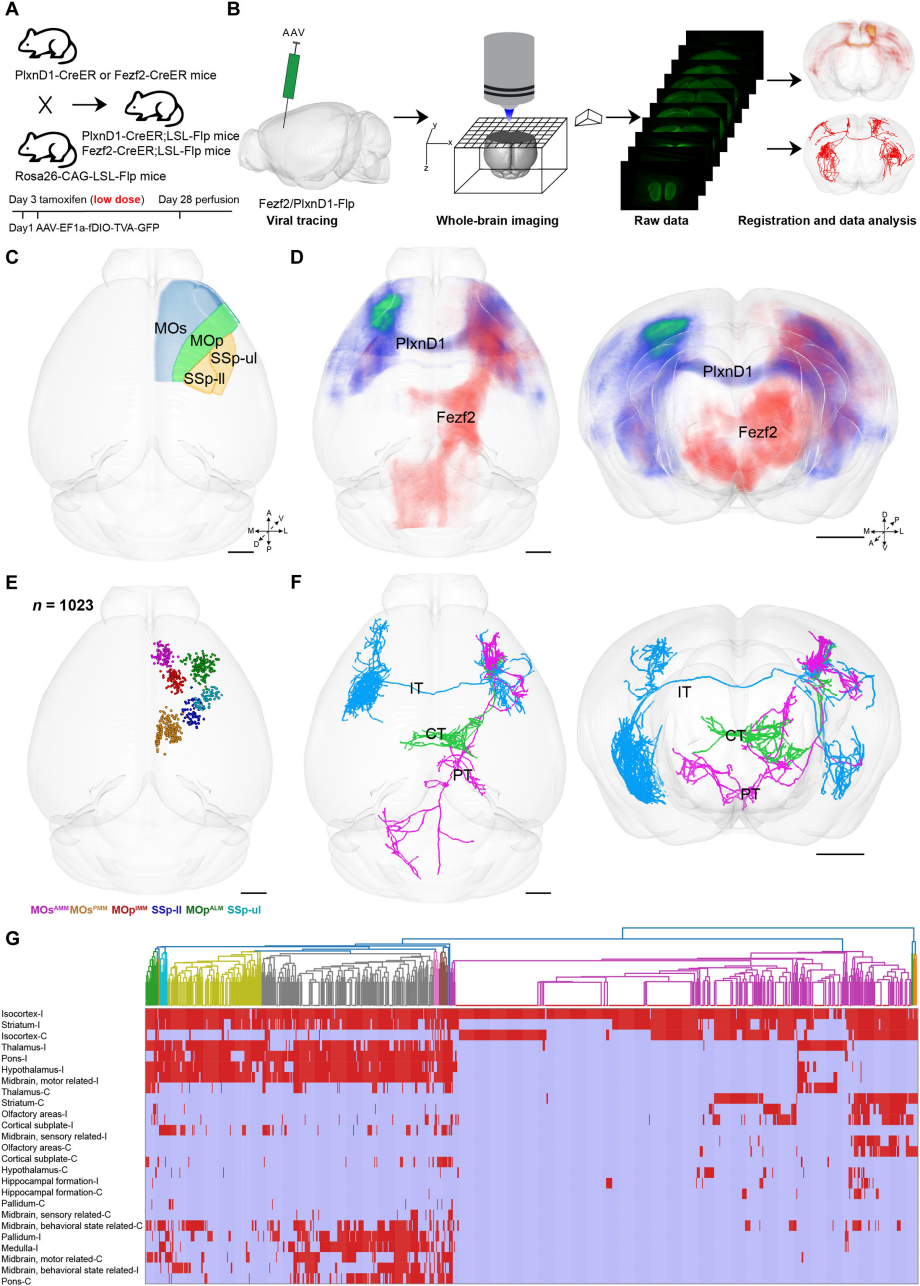
Whole-brain projection of cortical pyramidal neurons in the sensorimotor cortex. (A) Sparse labeling strategy. (B) Pipeline for analyzing the projection patterns of pyramidal neurons in the whole brain. (C) Spatial coverage of all injection sites. A, anterior; P, posterior; D, dorsal; V, ventral; M, medal; L, lateral. (D) The population-level projection pattern was derived from MOp^ALM^ using *Fezf2-2A-CreER* and *PlxnD1-2A-CreER* mice. (E) Three-dimensional distribution of reconstructed neuronal somas. The soma locations of 1,023 neurons from 6 subregions were presented in different colors. (F) Three exemplar neurons of different projection classes showed distinct projection patterns in the 3D brain outline. (C to F) Scale bars, 1 mm. (G) Clustering based on long-range projection targets of reconstructed neurons from the 6 subregions of the sensorimotor cortex. Each cluster division was colored differently on the dendrogram. For the projection matrix, columns represent single neurons and rows represent target regions. Red indicates axonal termination in the target region. I, ipsilateral; C, contralateral. The abbreviations of brain regions are provided in Table S1.

To examine the projection patterns at the single-neuron level, we labeled these pyramidal neurons sparsely and reconstructed the axonal projections of single neurons across the whole brain. We first crossed *Fezf2-2A-CreER* and *PlxnD1-2A-CreER* mice with *LSL-Flp* mice. After tamoxifen induction [23–26], the *Fezf2^+^* and *PlxnD1^+^* neurons start to express the Flp recombinase. We then injected anterograde Flp-dependent adeno-associated virus (AAV) to label these neurons with green fluorescent protein (GFP) [27] and validated high layer specificity of GFP*^+^* neurons in the 6 subregions [27] (Fig. 1A and Fig. S2A and B). Viral-injected brains were resin-embedded and imaged by the fMOST system with high spatial resolution at 0.35 × 0.35 × 1 μm^3^. The raw data were preprocessed and registered to the Allen Mouse Brain Common Coordinate Framework (CCF). Complete morphology of single neurons was reconstructed in Gtree software using continuous high-resolution dataset of the whole brain (Fig. S3A). The whole-brain datasets were analyzed using NeuroGPS [28,29] or Amira software (Fig. 1B). Based on the population-level projection pattern of different subregions of the sensorimotor cortex, it is unclear whether neuronal fibers pass through or terminate in a region and whether a target area receives input from the same group or different groups of neurons (Fig. 1C and D). Therefore, it is necessary to parse the complete morphology of single neurons. Using the morphological datasets with high resolution, we reconstructed 1,023 pyramidal neurons in the sensorimotor cortex from 70 brain samples (MOs^AMM^, *n* = 219; MOs^PMM^, *n* = 246; MOp^IMM^, *n* = 144; MOp^ALM^, *n* = 157; SSp-ul, *n* = 141; SSp-ll, *n* = 116) (Fig. 1E). Specifically, 530 and 493 neurons were acquired from *Fezf2-2A-CreER* and *PlxnD1-2A-CreER* mice, respectively. These pyramidal neurons encapsulated the major PT, IT, and corticothalamic (CT) classes and exhibited complex morphology with axons targeting multiple downstream areas through various routes [22] (Fig. 1F). Unsupervised hierarchical clustering of single-cell axons revealed 2 major projection types including a type with axons targeting multiple areas in the ipsilateral cortex, striatum, thalamus, pons, midbrain, pallidum, and medulla and another type with axons targeting fewer brain areas (Fig. 1G).

**Fig. 2.**
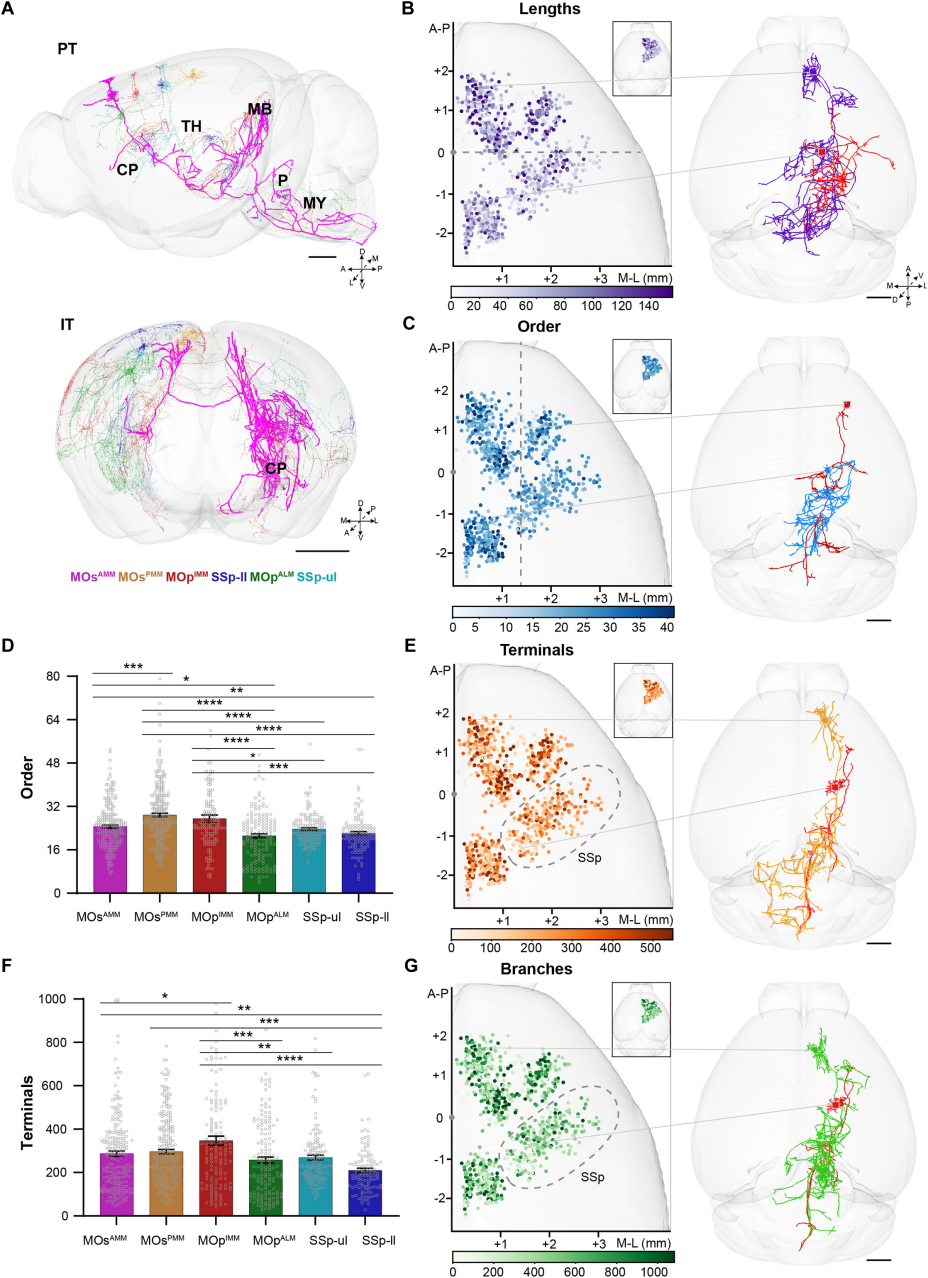
Extensive diversity of individual pyramidal neurons. (A) Complete morphology of exemplar reconstructed single neurons from the 6 subregions. Scale bars, 1 mm. (B and C) The length and order complexity of pyramidal neurons showed strong spatial location dependence. Scale bars, 1 mm. Circle represents the soma location of a neuron. The darker the color, the more complex the morphology. The complete morphology of exemplar complex and simple neurons is shown in 3D brain space. (D) Order of branches of pyramidal neurons in different subregions. One-way analysis of variance (ANOVA) followed by Tukey’s post hoc tests, *P < 0.05, **P < 0.01, ***P < 0.001, ****P < 0.0001. (E to G) The terminals and branches of pyramidal neurons in the motor cortex were more complex than those in the sensory cortex. Scale bars, 1 mm. (G) Comparison of total terminals.

The neuronal morphology was diverse in the same subregion, such as in MOp^ALM^. Some PT neurons projected to the zona incerta (ZI), superior colliculus (SC), and hypoglossal nucleus (XII) in the brainstem, while other PT neurons only projected to only some of these regions. The main projection routes of these reconstructed neurons were highly consistent with the population projection pattern, which indicates high accuracy of the reconstructed neuronal morphology (Fig. S3B). The reconstructed neurons cover multiple regions of the sensorimotor cortex and feature 3 major projection classes. The *Fezf2^+^* neurons were dominated by the PT type [22,30] sending fibers to multiple subcortical areas, whereas the *PlxnD1^+^* neurons were dominated by the IT type projecting bilaterally in the cortex and the striatum, with a proportion of more than 85% (Fig. 2A). In addition, we found a small proportion of IT- and CT-type neurons within the *Fezf2^+^* population and a few PT-type neurons within the *PlxnD1^+^* population (Fig. S3C).

The morphological characteristics, including the number of terminals, total lengths, and the number and order of branches, reflect the complexity of 1,023 pyramidal neurons and are distinct in different regions. Compared with those in the posterior regions, the total length of axons for neurons in the anterior regions was substantially larger (Figs. S4 to S6). The total length of axons was closely related to the position of the soma along the anterior/posterior axis (Fig. 2B and Figs. S4 to S6). The length of the axons of individual neurons in the anterior subregions (except for MOp^ALM^) was significantly larger than that of individual neurons in SSp-ll (*P* < 0.05; Fig. S7A). Neurons in the medial regions had more complex branches than those in the lateral regions. The order of axon branches of pyramidal neurons was closely related to the soma position along the medial/lateral axis (Fig. 2C and Figs. S4 to S6). The order of branches of pyramidal neurons in the medial subregions including MOs^AMM^, MOs^PMM^, and MOp^IMM^ was significantly higher than that of pyramidal neurons in the lateral subregions including MOp^ALM^, SSp-ul, and SSp-ll (*P* < 0.05; Fig. 2D). In particular, the order of branches from neurons in MOp^ALM^ was significantly lower than that from neurons in MOp^IMM^ (*P* < 0.0001; Fig. 2D).

Furthermore, we found that the axonal terminals and branches of the neurons in the motor cortex were more abundant than those of the neurons in the sensory cortex, irrespective of the PT or IT type (Fig. 2E to G and Figs. S4 to S6). The number of total terminals and branches of neurons in MOs^AMM^, MOs^PMM^, and MOp^IMM^ were significantly larger than that of neurons in MOp^ALM^, SSp-ul, and SSp-ll (*P* < 0.05; Fig. 2F and Fig. S7B). In SSp-ll, the number of total terminals and branches of neurons was significantly lower than that of neurons in the motor subregions except for MOp^ALM^ (*P* < 0.05; Fig. 2F and Fig. S7B). Additionally, neurons in MOp^ALM^ had significantly greater number of total terminals and branches than those in MOs^PMM^ (*P* < 0.001; Fig. 2F and Fig. S7B). In summary, our results suggest that morphological features of single neurons are highly related to the soma location across cortical areas.

Our data further reveal a topographical organization of the axonal projections of PT and IT neurons in different subregions, especially in the target areas of SC and caudoputamen (CP). The summed axon termination patterns of all reconstructed single neurons are similar to those reported previously using mesoscale tracing [9] (Figs. S7C and S8).

### Morphological diversity of PT neurons

**Fig. 3.**
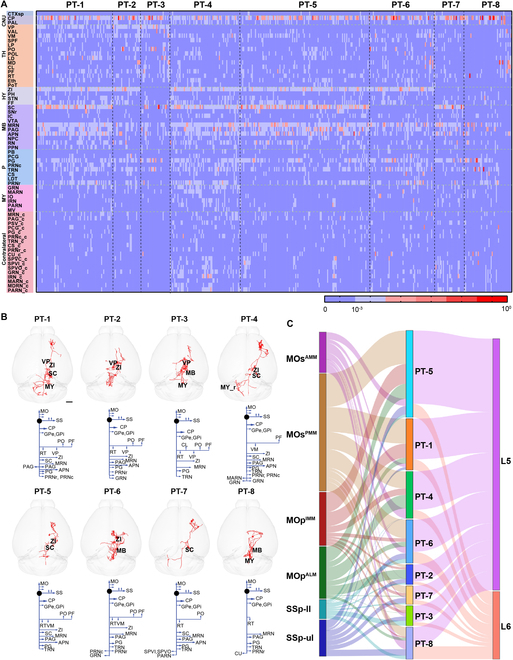
Long-range projection subtypes of PT neurons at the single-cell level. (A) Heatmaps of projection matrix of PT neurons. Each column shows the normalized terminals of one individual neuron in different targeting areas. Color represents the percentage of the terminals in a specific targeting area over total terminals. Based on the terminal’s location and the strength, the PT neurons were grouped into 8 types. The abbreviations of brain regions are provided in Table S1. (B) Schematics of representative projection patterns of different PT subtypes. Scale bar, 1 mm. (C) Relationship between PT subtype and areal/layer location of neuronal somas.

The PT neurons extend their axons to multiple subcortical brain regions, including the thalamus, midbrain, pons, medulla oblongata, and spinal cord (Fig. 3A). Compared with the IT neurons, PT neurons were more heterogeneous in their axon termination patterns. Individual PT neurons targeted various subcortical regions with distinct preferences for specific nuclei including CP, ZI, and SC (Fig. 3A). To compare the projection pattern of individual neurons, we normalized the total terminal number across the whole brain and calculated the percentage of the terminal number in specific targeting areas. Based on the distribution of terminals in different brain regions, we identified 8 subtypes across all reconstructed PT neurons (*n* = 448). Neurons of cluster PT-1 to PT-3 projected to the ventral posterior complex of the thalamus (VP), whereas neurons of other clusters (PT-4 to PT-8) did not. VP plays key roles in cortico-thalamo-cortical loops and processes somatosensory, especially nociceptive information [31,32]. Thus, we employed VP as an important projection target to drive cluster segregation [10].

Each PT subtype had its preferred target areas. Compared with PT-2, which projected to the ipsilateral ZI and VP, PT-1 additionally projected to SC, whereas PT-3 only projected to VP and had few terminals in the contralateral hemisphere. Although the neurons of PT-4 to PT-8 projected to multiple ipsilateral and contralateral regions, they did not project to VP. PT-7 to PT-8 did not project to ZI. PT-6 and PT-8 did not project to SC. Compared with the neurons of PT-4, the neurons of PT-5 avoided some regions in the medulla, such as the gigantocellular reticular nucleus (GRN) and the magnocellular reticular nucleus (MARN) (Fig. 3A and B).

We wondered whether different PT subtypes correspond to specific layers or subregions of the cortex. By comparing the soma location of neurons in different subtypes, we found that the PT subtypes exhibited areal preference. MOs^PMM^ and MOp^IMM^ incorporated abundant neurons of the PT-1 and PT-5 subtypes that all target ZI (Fig. 3C and Table 2). MOp^ALM^ consisted of neurons of the PT-5 and PT-7 subtypes, which all innervate SC. In SSp-ul and SSp-ll, a similar number of neurons of different PT subtypes (PT-8, PT-3, PT-6, and PT-5) were found. In addition, these PT neurons mainly reside in layer 5 with a few in layer 6 but not in layer 2/3, consistent with previous studies [22,30] (Fig. 3C).

**Fig. 4.**
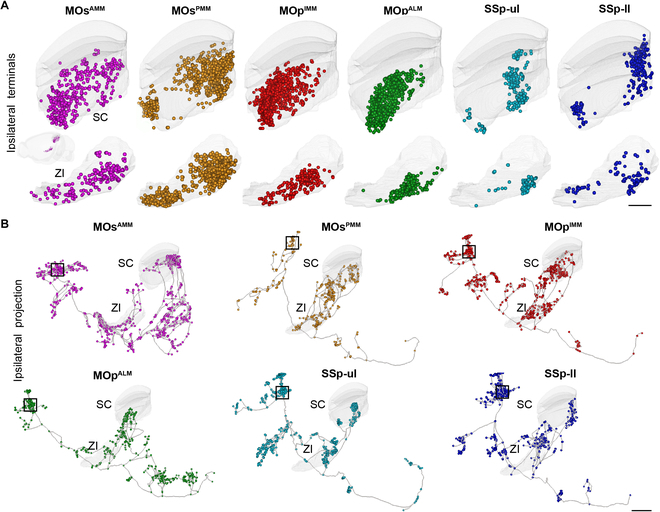
The distribution of axonal terminals from PT neurons in the ipsilateral SC and ZI. (A) Axonal distribution patterns of PT neurons in the ipsilateral SC and ZI. Circles represent axonal terminals from a single neuron. Scale bar, 500 μm. (B) Whole-brain long-range projection of exemplar PT neurons. The black rectangle indicates the location of the neuronal somas. Scale bar, 1 mm.

Notably, most PT neurons in different subregions of the sensorimotor cortex project to the ipsilateral SC and ZI (Fig. 3A). By examining their axonal terminals in the ipsilateral SC and ZI, we found that PT neurons in the same subregion showed similar dominant projection patterns, while the patterns of neurons in different subregions were significantly different. Specifically, the axonal terminals of PT neurons in MOs^AMM^ were dispersed in the ventral region of SC and ZI. The axonal terminals of PT neurons from MOp^IMM^ and MOp^ALM^ were concentrated in the anteroventral region, while those from the other 3 subregions formed 2 separate clusters, distributed in the anteroventral and posteroventral part. Compared with SSp-ul, the axonal terminals of PT neurons in SSp-ll distributed more toward the posterior region (Fig. 4A and Fig. S9A). However, at the population level, the topological output patterns of these PT neurons in SC and ZI could not be clearly distinguished due to the interference of passing fibers (Fig. S9B). To examine thalamic targeting of PT and CT neurons, we compared the number of thalamic nuclei that receive axonal input from these neurons. We found that CT neurons form circuit connections with more thalamic nuclei than PT neurons (Fig. S9C). However, PT neurons that sent collaterals to the thalamus showed more complex morphological features, including total axon lengths, order of axonal branches, and number of terminals and branches (Fig. S9D).

By scrutinizing the dominant projection patterns in SC and ZI, we found that PT neurons of different subregions had various projection patterns, which were reflected in the different combinations of the projection targets. Only a few PT neurons had projection pattern matching observed at the population level (Fig. 4B). These results highlight the need to reveal the fine projection patterns of neurons at the single-cell level.

### Morphological diversity of IT neurons

**Fig. 5.**
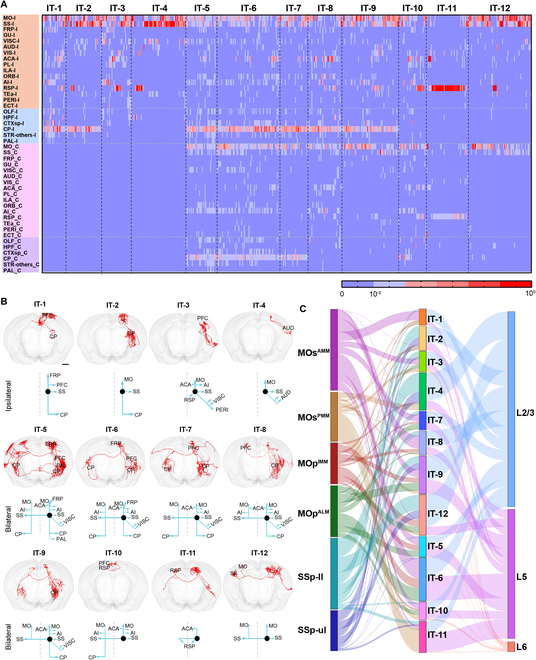
Morphological analysis of cortical IT neurons. (A) Heatmaps of projection matrix of IT neurons. Color indicates normalized number of terminals in each target area. The abbreviations of brain regions are provided in Table S1. (B) Schematics of representative projection patterns of different IT subtypes. Scale bar, 500 μm. (C) Relationship between IT subtype and areal/layer location of neuronal somas.

We reconstructed the fine morphology of 536 IT neurons from the 6 subregions. Although most of these IT neurons mainly projected bilaterally to the striatum and cortical regions, some of them had axonal branches in other regions, such as the anterior olfactory nucleus, piriform area, postsubiculum, endopiriform nucleus, fundus of the striatum, and pallidum (Fig. 5A). The reconstructed IT neurons had diverse morphological characteristics including the targeting areas, the number and density of axonal terminals, the axonal branches, and the projection routes. To classify the projection patterns of the IT neurons, we quantified the axonal terminals of individual neurons in different targeting regions and subsequently grouped these neurons into 12 subtypes with different connection preferences (Fig. 5A). IT-1 to IT-4 mainly projected to ipsilateral brain regions instead of contralateral regions, whereas IT-5 to IT-12 all had axonal terminals in the contralateral cortex. A previous study has shown that the ipsilateral and contralateral projections regulate different functions in mice [33]. Thus, it is imperative to analyze the fine structures of these projections.

Each subtype shared a set of common targets and had multiple axonal branches that targeted different downstream areas. IT-1 , IT-2, and IT-5 to IT-9 had rich axonal branches in the ipsilateral CP. IT-5 to IT-7 and IT-10 had branches in the contralateral CP (Fig. 5B). Additionally, most IT subtypes projected to the anterior cingulate, prelimbic, and infralimbic areas of the prefrontal cortex (PFC) except IT-2, IT-4, and IT-12. Furthermore, there are neurons in some subtypes projecting to the frontal pole, contralateral PFC, and pallidum (Fig. 5A), which indicates that they may participate in different functional circuits.

We wondered whether these different IT subtypes correspond to different cortical regions and/or layers. With the cytoarchitectonic information, we confirmed the soma location of individual neurons in the 3-dimensional (3D) space and found that the 6 subregions consisted of different combinations of the 12 IT subtypes (Fig. 5C). Furthermore, the proportion of these 12 subtypes was different in the 6 subregions. MOs^AMM^, MOp^IMM^, and MOp^ALM^ were dominated by IT-6, whereas MOs^PMM^ mainly contained the IT-11 neurons, which preferentially targeted the retrosplenial area bilaterally with minor projections to other cortical regions (Fig. 5C). These results indicate that IT neurons in the posterior subregions have a limited number of targets compared with those in the other parts of the motor cortex. There were fewer IT subtypes in the sensory cortex compared to the anterior motor cortex including MOs^AMM^, MOp^IMM^, and MOp^ALM^ (Fig. S9E and Table 3). The neurons of the sensory cortex mainly belonged to IT-2, IT-4, and IT-12. While SSp-ul contained IT-8, SSp-ll was dominated by IT-9. These results indicate that the higher-level cortices employ a wide variety of IT subtypes to perform more diverse and complex functions.

The reconstructed IT neurons were mainly distributed in layers 2/3 and 5 with a few in layer 6 (Fig. 5C). Some subtypes showed a clear layer preference. For example, more than half of the neurons in IT-1 to IT-4, IT-7 to IT-9, and IT-12 were located in layer 2/3. We further found that the IT neurons in layer 5 targeted more downstream regions than those in layer 2/3, which suggests more complicated projection patterns of IT neurons in layer 5. The IT-1 to IT-4 neurons restricted their axon terminals in the ipsilateral telencephalon, while the IT-7 to IT-9 neurons projected broadly to the ipsilateral cortical areas, CP, and the contralateral sensory areas. Although the neurons of IT-5, IT-6, IT-10, and IT-11 were found more in layer 5, the other subtypes except IT-11 innervated more downstream targets (Fig. 5C).

To further explore the relationship between soma location and morphological properties of individual neurons, we compared the IT neurons located in different layers. The morphological characteristics of these 536 IT neurons were all obtained from the neurons shown in Fig. 2B to G. IT neurons in layer 5 demonstrated more complex projection patterns than those in layer 2/3 (Fig. S10A), while the number of total terminals and branches did not show significant difference (Fig. S10B and C). On the contrary, the total length of the axons of the layer 5 IT neurons in MOs^PMM^ and MOp^ALM^ were significantly larger than those of the layer 2/3 IT neurons, whereas the order of axon branches of layer 2/3 IT neurons was notably higher than that of layer 5 IT neurons, especially in SSp-ll and SSp-ul (Fig. S10D and E).

Given the bilateral projection of some IT neurons, we next focused on the contralateral cortical projections of IT neurons in different layers. We normalized the number of terminals in each layer based on the total number of terminals in the contralateral cortex. We found that the contralateral cortical axon terminals of IT neurons from layers 2/3 and 5 of different subregions were mainly distributed in layers 1, 2/3, 5, and 6 and were almost absent in layer 4 (Fig. S10F). Interestingly, the contralateral cortical axon terminals of IT neurons from the motor cortex including MOs^AMM^, MOs^PMM^, MOp^IMM^, and MOp^ALM^ were more distributed in layer 1 than those from the sensory cortex. Conversely, IT neurons in layers 2/3 and 5 of SSp-ul and SSp-ll had more axonal terminals in layer 6 of the contralateral cortex compared to those of the motor cortex (Figs. S10F and S11A). IT neurons in the motor and sensory cortices have a considerable number of axon terminals in the layer 5 of the contralateral cortex (Fig. S10F). These results reveal clear laminar innervation patterns in the contralateral cortex and suggest that the IT neurons in the motor cortex have major feedback regulation effect on the contralateral cortex, while the IT neurons in the sensory cortex have both feedforward and feedback effect. Overall, these results again confirm that the projection patterns of single neurons are highly correlated with the soma locations.

In addition, we found that the *Fezf2* marker gene captures a few IT-type neurons (Fig. S3C). The *Fezf2^+^* IT neurons exhibited similar morphological characteristics as the *PlxnD1^+^* IT neurons, including the number of targeted nuclei, total axon lengths, order of axon branches, and number of axon terminals and branches (Fig. S11B and C). In conclusion, the diverse morphological features of IT neurons are correlated with their areal and layer locations.

### Single-neuron projectome

In summary, cortical pyramidal neurons can be classified into multiple projection subtypes based on their diverse axonal targets. In addition, the distribution of PT and IT subtypes in different subregions and layers showed a certain degree of preference. These cellular-level results suggest the existence of cortical subnetworks, which is crucial for the understanding of cortical circuit organization.

**Fig. 6.**
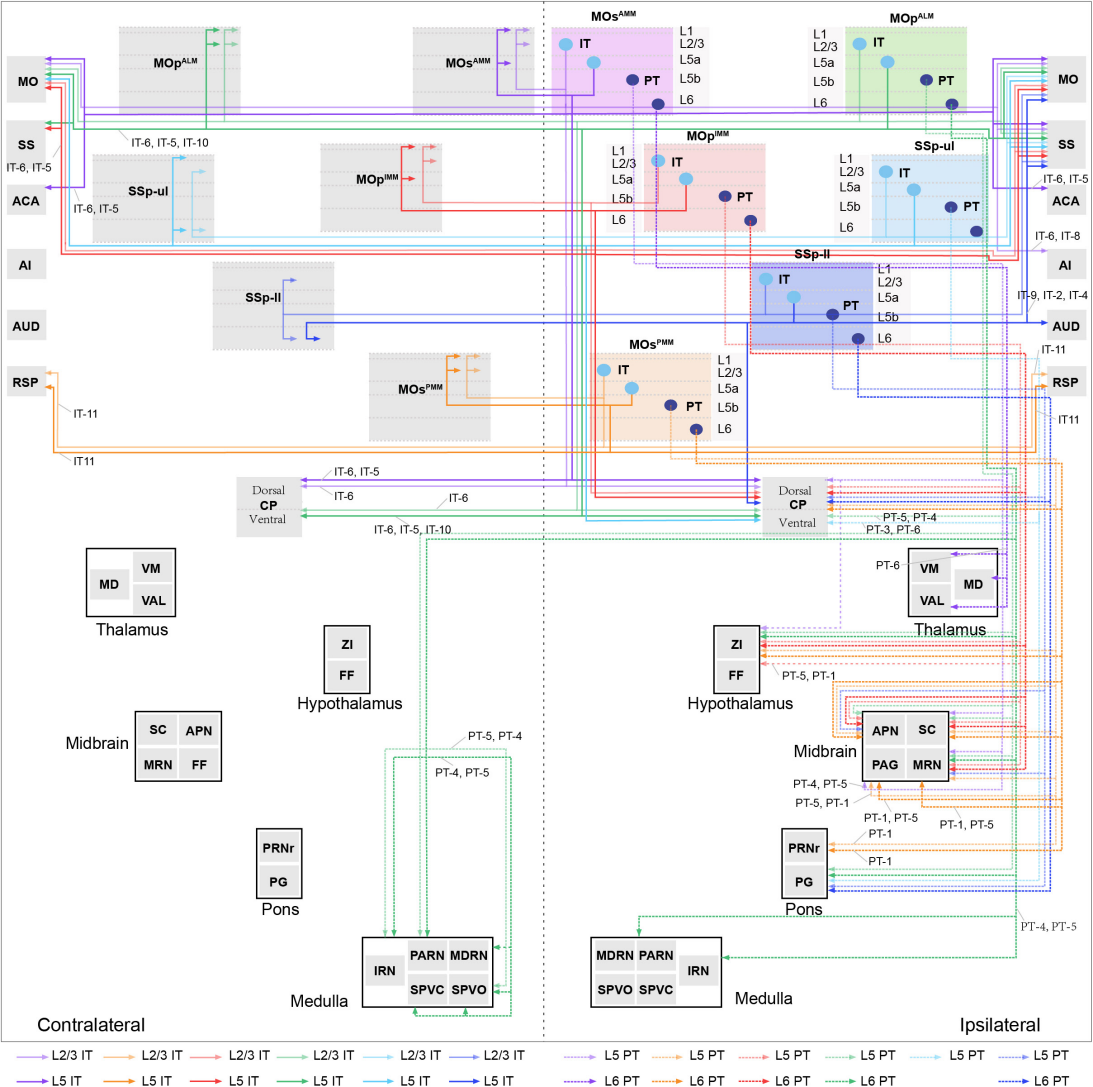
Wiring diagram of cortical PT and IT neurons in different layers. Gray boxes depict projection targets. Colored boxes indicate distinct source areas. Projections with connection strength over 0.05 were shown. Connection strength is the ratio of axonal terminals in a target area over all axonal terminals.

The precise morphological characterization of 1,023 neurons reveals some basic projection features of cortical pyramidal neurons. PT neurons in layer 5 preferentially innervated the ipsilateral CP more than those in layer 6 (Fig. 6). PT neurons in different subregions of the sensorimotor cortex selectively targeted different subcortical nuclei. For example, PT neurons in layer 6 of MOs^AMM^ preferentially regulated the ipsilateral thalamus, and those of MOp^ALM^, MOp^IMM^, and MOs^PMM^ tended to innervate the ipsilateral ZI. PT neurons in MOs^PMM^ innervated more in the periaqueductal gray (PAG), midbrain reticular nucleus (MRN), and pontine reticular nucleus (PRNr) than those in the other subregions, and those in MOp^ALM^ tended to project bilaterally in the medulla (Fig. 6).

IT neurons in different layers of the motor cortex preferentially send axons in layers 1, 2/3, and 5a of the contralateral cortex, while those of the sensory cortex preferentially send projection in layers 1, 2/3, and 6. Compared with layer 5, IT neurons in layer 2/3 tended to project more to the ipsilateral CP (Fig. S11D). The axonal terminals in the contralateral CP were mainly derived from IT neurons in MOs^AMM^ and MOp^ALM^. Distinct from IT neurons in other subregions, the IT neurons in MOs^PMM^ mainly project bilaterally to the retrosplenial area (RSP) (Fig. 6).

MOp^ALM^ and MOp^IMM^ both incorporated PT-5, with differences reflected in the proportion of PT-1, PT-4, and IT-7. SSp-ul and SSp-ll shared the subtypes of PT-6 and PT-8, with differences in the proportion of PT-3 and PT-5. Compared with the motor cortex, the sensory cortex had a larger proportion of PT-6 and PT-8 subtypes, which strongly innervate the ipsilateral CP, ZI, and multiple midbrain regions. On the other hand, the primary motor cortex had a larger proportion of PT-1 neurons, which regulated the ipsilateral CP, ZI, SC, and VP (Fig. 6 and Fig. S12).

Regarding the IT subtypes, MOp^ALM^ and MOp^IMM^ shared the subtypes of IT-5, IT-6, and IT-12 with neurons equally distributed in layers 2/3 and 5, whereas they had different proportions of IT-9 and IT-7 neurons that are primarily located in layer 2/3. SSp-ul and SSp-ll shared the subtypes of IT-2, IT-4, and IT-12 with neurons equally distributed in layers 2/3 and 5, whereas they had a different proportion of IT-9 neurons that are primarily located in layer 2/3. Interestingly, the primary sensory cortex incorporated more neurons from IT-2 and IT-4, which regulate multiple ipsilateral cortical regions, whereas the primary motor cortex contained more neurons from IT-5 and IT-6, which modulate the bilateral sensorimotor cortex, PFC, CP, and the ipsilateral pallidum (Fig. 6 and Fig. S12). In summary, our connectivity data provide a framework for understanding how different cortical projection types in the sensorimotor cortex participate in global brain circuits that process complex sensory information and generate proper adaptive motor output accordingly.

## Discussion

Employing transgenic mice, sparse labeling, and the fMOST system, this study reveals the complete morphology of 1,023 pyramidal neurons from 6 subregions of the sensorimotor cortex. Morphological characteristics of IT and PT neurons in different subregions and layers were further analyzed, including the total axon lengths, the number of axonal terminals and branches, and the order of axonal branches. The different morphological characteristics were closely related to the neuronal soma position in 3D space. Based on the distribution of axon terminals in distinct downstream target regions, IT and PT neurons were classified into various subtypes. These subtypes of neurons with different projection patterns had unique regional and layer distribution characteristics. Together, our results reveal major projection and morphological differences across layers and cortical regions, as well as individual neuron-to-neuron variations within each projection subtype.

The classification of neuronal types has been based on several features: intrinsic electrophysiological properties, morphological characteristics, gene expression profiles, and connectivity patterns [11,34]. Although the long-range projection pattern of a single neuron may not perfectly correspond to its transcriptomic type, previous findings illustrated the importance of revealing the diversity and specificity of neuron morphology and projection at the single-cell level [10]. Compared with axonal tracing at the population level [30], axonal reconstruction of single neurons avoids the interference of passing fibers and thus could better reflect the true connectivity between brain regions. However, this method is less accurate than the synaptic labeling method that can reveal the strength of connection between different brain regions [35,36].

Compared with the sensory cortex and lateral motor cortex, the neurons in the medial motor cortex were more complex in the terminal and branch number, and the order of axonal branches (Fig. S13). This neuronal morphological difference between the somatosensory and the motor cortex is consistent with the differential positions of these regions in the hierarchical cortical network [30]. Our results further unveil the morphological differences between higher- and lower-order cortices and the complexity of motor areas.

The projection patterns of pyramidal neurons correlated with the soma location in subregions and layers. Topographic correspondence between soma locations and major axon arbors was observed in all projection types in the 6 subregions, which is in line with a recent study [10]. The axonal terminals of the IT neurons in layer 2/3 of the sensory cortex were concentrated in layers 2/3 and 6 of the contralateral cortex, while those of the IT neurons in layer 5 were skewed to layers 1 and 6. By reconstructing the morphology of 287 IT and PT neurons in the motor and sensory cortices, a recent study has shown that the distal axon terminals of layer 2/3 IT neurons in the sensory cortex are concentrated in the middle layers (layer 2/3 to 5), whereas the axon terminals of layer 5 IT neurons preferentially target layer 1 [10]. These results indicate that the IT neurons in layer 2/3 of the sensory cortex bilaterally regulate the activity of the cortex in a feedforward manner, and the IT neurons in layer 5 have feedforward effects on the ipsilateral cortex and both feedforward and feedback effects on the contralateral cortex [37,38]. The axon terminals of IT neurons in layers 2/3 and 5 of the motor cortex were concentrated in layers 1 and 2/3 of the contralateral cortex, echoing a recent study showing that the distal axon terminals of IT neurons in the motor cortex are concentrated in layer 1 [10]. These results indicate that IT neurons in the motor cortex exert feedback effects on both ipsilateral and contralateral cortices. This projection-type difference between the somatosensory cortex and motor cortex is consistent with the different positions of these regions in the hierarchical cortical network [30].

It is noteworthy that deep-layer PT neurons in MOp^ALM^ had more axonal terminals distributed in the medulla than those in the other subregions. The PT neurons in layer 5 projected bilaterally to the medulla, whereas the PT neurons in layer 6 only projected to the contralateral medulla. These results reveal unique and complex medulla projections of neurons in MOp^ALM^, as opposed to the extensive projections of neurons in the MOp [39], highlighting the importance of cross-regional anatomy of specific cell types.

Previous studies have shown that different subregions are involved in regulating different functions, which is closely related to the efferent connections of neurons [5,40]. Our 6 subregions were composed of PT neurons of different subtypes mainly distributed in layer 5 with a few in layer 6. MOp^IMM^ has a high percentage of PT-1 neurons, which project to VP and may play an important role in sensory–cognitive functions [41,42]. MOp^ALM^ contained a high proportion of PT-4 and PT-7 neurons, which project to multiple subcortical regions, including the ipsilateral CP, and thalamic regions except for VP, ZI, pons, and bilateral midbrain and medulla. This multi-area projection in the subcortical regions may be responsible for its diverse functions [12,39,43–45]. On the contrary, SSp-ul and SSp-ll incorporated a high proportion of PT-5 neurons, which strongly project to CP, ZI, and SC, of which ZI is the site of sensory integration and regulates sensory transmission [46,47], while SC is involved in the motor and sensory functions of the forelimb [48]. SSp-ul also had many PT-3 neurons regulating multiple thalamic regions including VP, which may explain its distinct function compared to SSp-ll [41]. PT neurons in the same region exhibited similar projection patterns in SC and ZI. Recent studies showed that the connection between ZI and SC was topologically organized [47,48]. This further suggests that the PT projections to SC and ZI may be involved in similar functions of sensory and motor processing.

IT neurons play various roles in the processing of the same sensory stimulus [1,49,50], which highlights the different functional roles of different IT subtypes and the importance of their integration. Compared with MOp^IMM^, MOp^ALM^ contained more IT-9 and IT-7 neurons, which project to similar downstream targets, and thus may participate in similar functions [1,51,52]. SSp-ll incorporated a larger proportion of neurons from IT-9 than SSp-ul. The IT-9 neurons regulate similar targets including the bilateral sensory and motor cortices, and the ipsilateral CP, that are responsible for the transmission and processing of sensory information [6,53]. Compared with the primary sensory cortex, the major IT neuronal subtypes included in the primary motor cortex innervated more downstream target areas, which may underlie the complex functions of MOp involving the execution of higher-order functions [54,55]. In addition to the bilateral cortex and striatum, IT neurons in the sensorimotor cortex had other projection targets, including the anterior olfactory nucleus, piriform area, postsubiculum, endopiriform nucleus, fundus of the striatum, and pallidum, which is consistent with other studies in the sensory cortex, motor cortex, and PFC [10,11,56].

To summarize, by single-neuron morphological reconstruction, this study reveals the hierarchical, layer, region, and cell type-specific connection patterns of cortical circuits. IT neurons in the motor cortex have predominantly feedforward projection to regulate the contralateral cortex, whereas those in the sensory cortex have both feedforward and feedback projections. IT and PT neurons from different subregions of the sensorimotor cortex establish distinct sets of subcircuits, which may underlie the complex and diverse functions of the sensorimotor cortex, including sensory perception and motor planning and execution [11]. Future studies may incorporate sex-difference analyses to investigate potential disparities in the neural circuits of sensorimotor cortex between genders.

## Data Availability

All data necessary to evaluate the conclusions of this study are included in the paper and the supplementary materials. Additional data related to this paper may be requested from the author upon reasonable request.

## Supplementary Materials

Supplementary 1 Figs. S1 to S13 Tables S1 to S3 Materials and Methods References [57–59]
