# Supplementary material for: From Individual to Population: Circuit Organization of Pyramidal Tract and Intratelencephalic Neurons in Mouse Sensorimotor Cortex
Source: Research (Wash D C). 2024 Oct 7;7:0470. doi: 10.34133/research.0470 (PMC11456696; doi:10.34133/research.0470)
Supplement: Supplementary 1 — Figs. S1 to S13 Tables S1 to S3 Materials and Methods References [57–59] [file research.0470.f1.zip › Supplemental materials.docx]

**Supplemental materials**


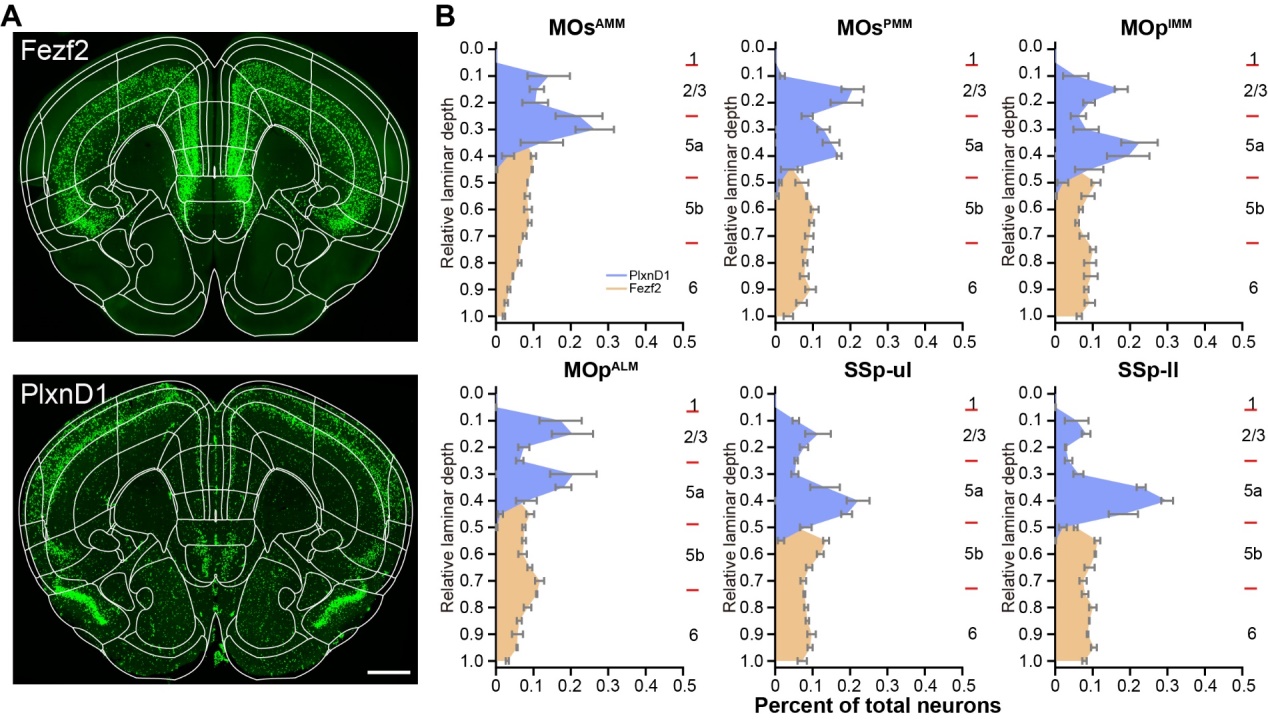
**Supplementary Fig. 1 | Laminar distribution of pyramidal neurons labeled using transgenic driver mice.** A, Layer distribution of *Fezf2+* and *PlxnD1+* neurons in the cortex. *Fezf2/PlxnD1-CreER* mice were crossed with *Rosa26 Tcf/Lef-LSL-H2B-GFP* mice to obtain the cortical distribution patterns. Scale bar, 500 µm. B, Layer distribution of *Fezf2+* and *PlxnD1+* neuronal soma in different subregions of the sensorimotor cortex. Data were derived from three samples of two mice. Error bars, ± s.e.m.


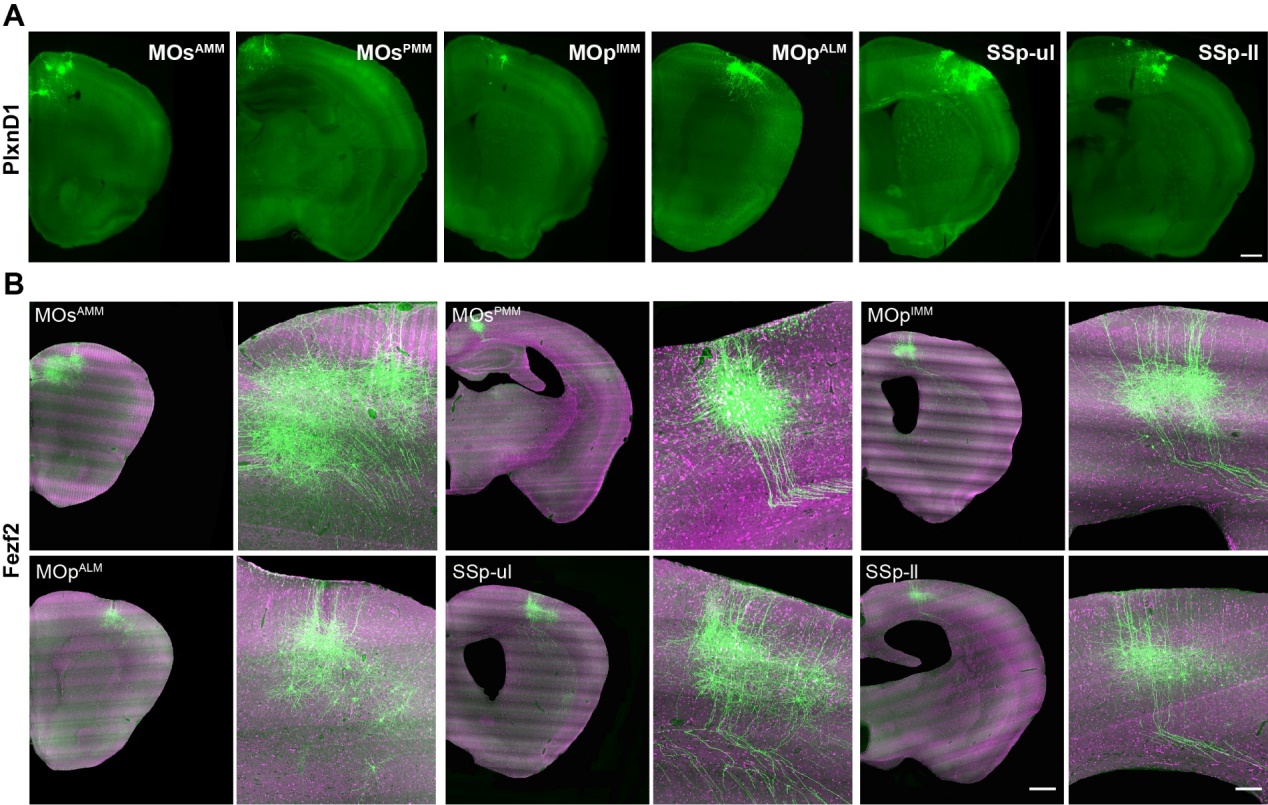


**Supplementary Fig. 2 | Labeling of *Fezf2+* and *PlxnD1+* neurons in the six subregions.** A, The injection sites of *PlxnD1* mice. Scale bar, 500 µm. B, The injection sites of *Fezf2* mice. The scale bars on the left and right indicate 500 and 100µm respectively.


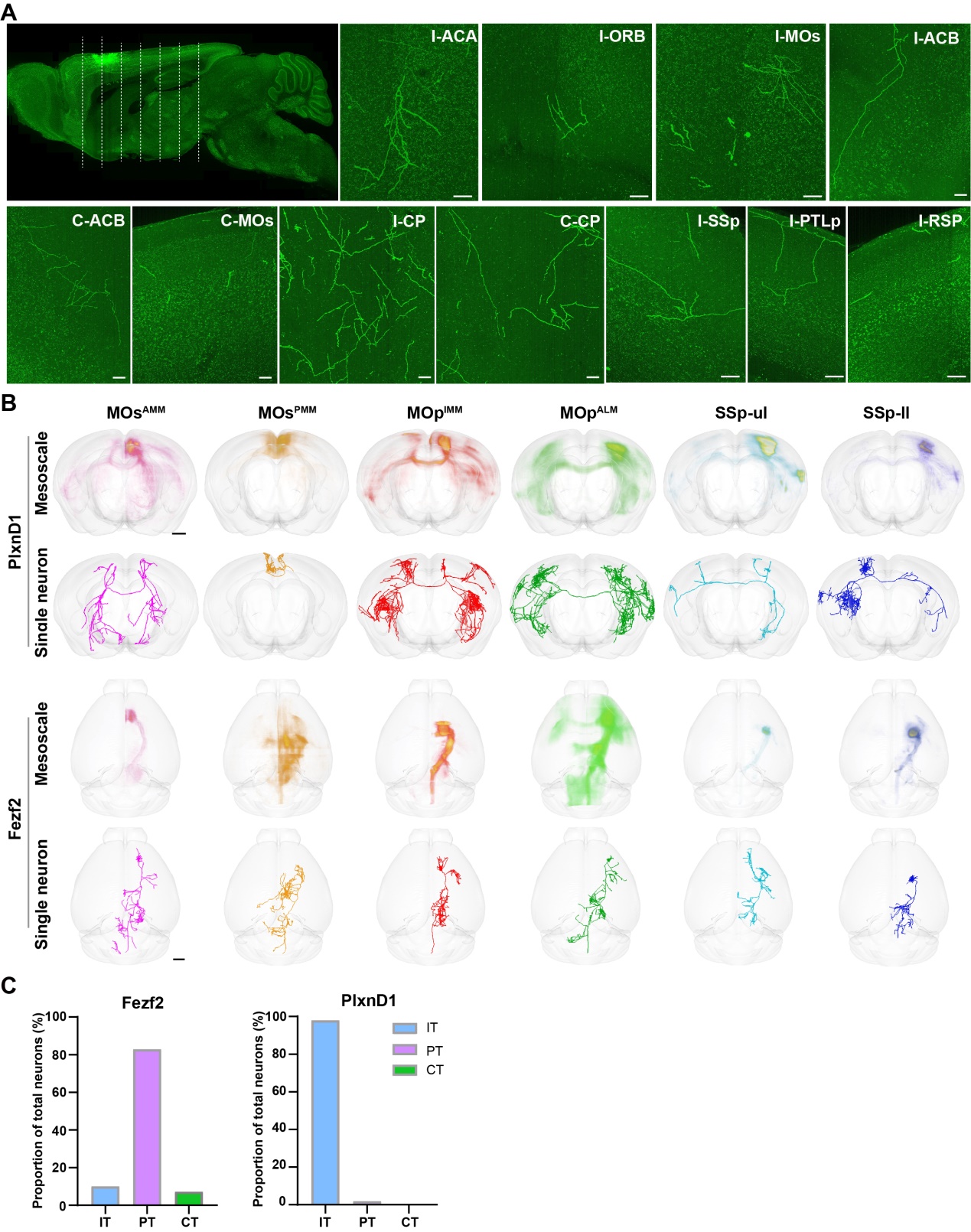


**Supplementary Fig. 3 | Long-range projection of pyramidal neurons at the single-cell level.** A, The tracing of single-neuron axons across the entire brain. Scale bars, 100 µm. ‘C’ and ‘I’ represent contralateral and ipsilateral respectively. The abbreviations of brain regions are provided in Supplementary Table 1. B, Comparison of projection patterns between single neuron and neuronal population in different subregions. *PlxnD1* pannels: Scale bar, 500 µm. *Fezf2* pannels: Scale bar, 1 mm. C, Proportions of *Fezf2+* and *PlxnD1+* neurons in different projection classes.


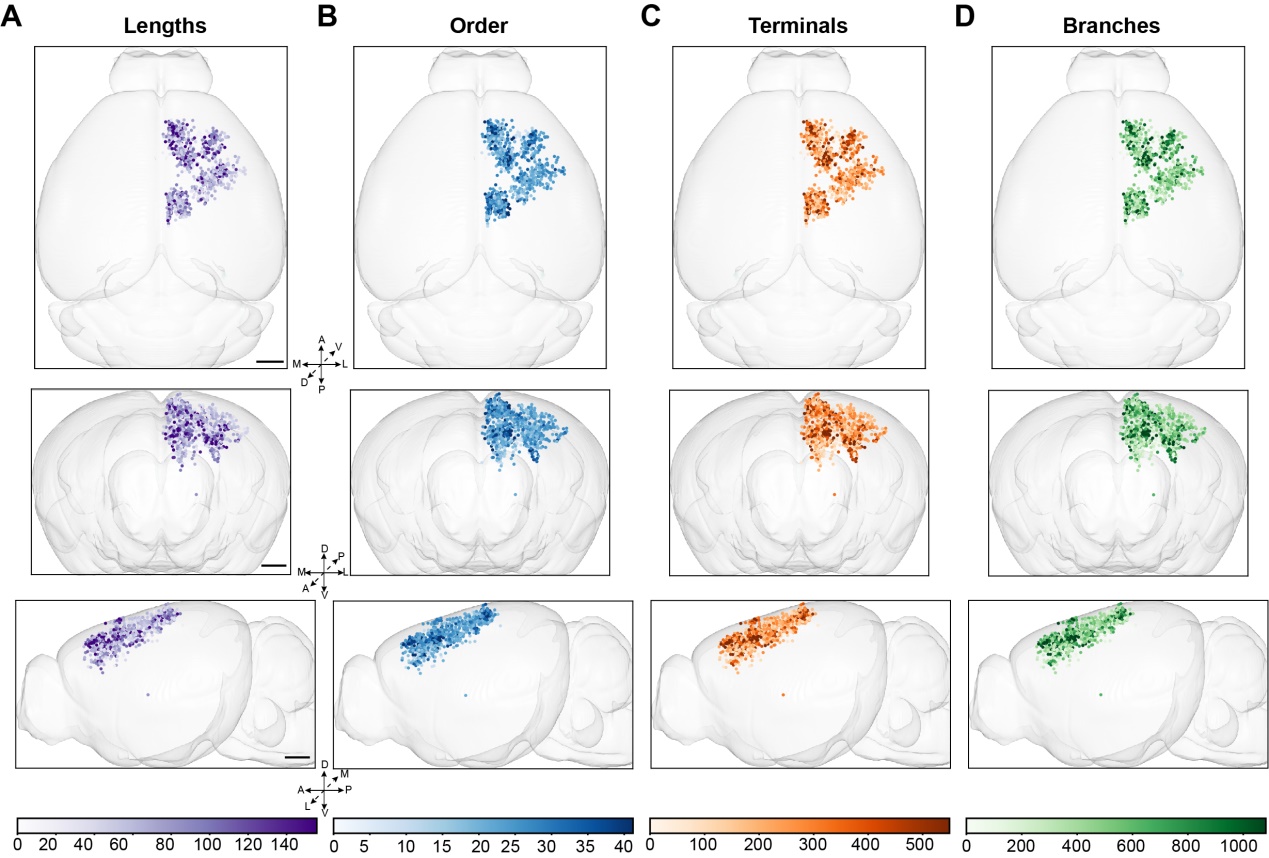


**Supplementary Fig. 4 | The relationship between morphological complexity and soma location of all reconstructed pyramidal neurons.** A, Lengths. Scale bars, 1 mm. B, Orders. C, Terminals. D, Branches. Each dot represents a single neuron.


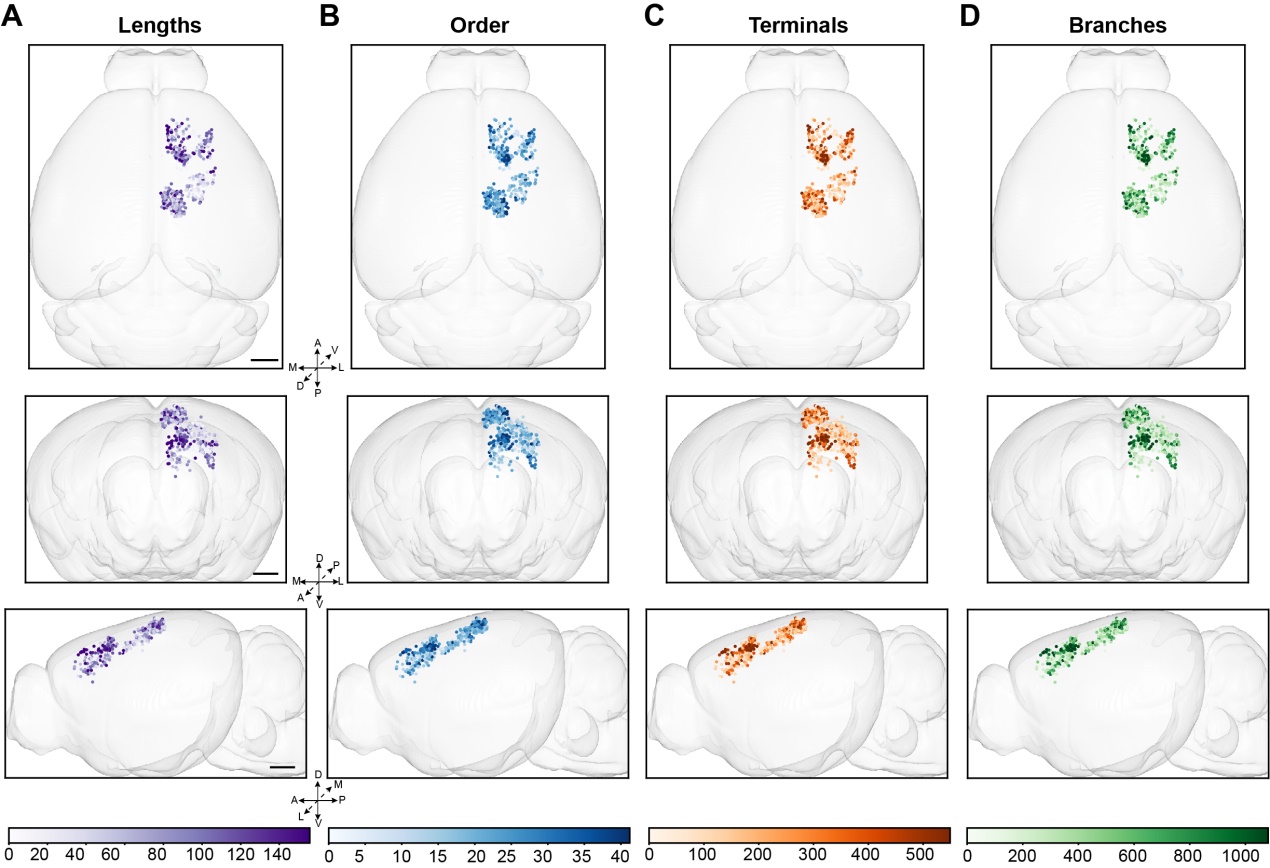


**Supplementary Fig. 5 | The relationship between morphological complexity and soma location of reconstructed PT neurons.** A, Lengths. Scale bars, 1 mm. B, Orders. C, Terminals. D, Branches. Each dot represents a single neuron.

**
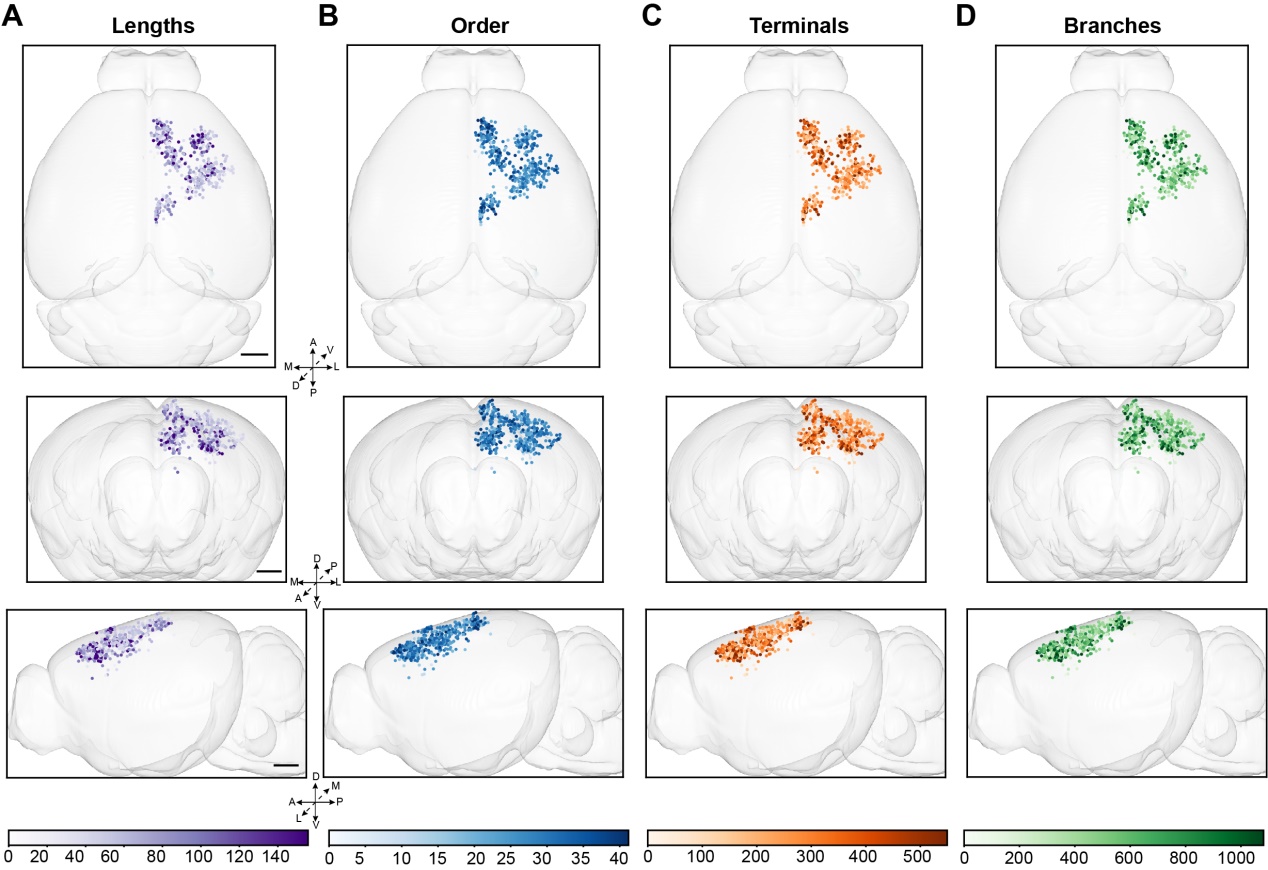
**

**Supplementary Fig. 6 | The relationship between morphological complexity and soma location of reconstructed IT neurons.** A, Lengths. Scale bars, 1 mm. B, Orders. C, Terminals. D, Branches. Each dot represents a single neuron.


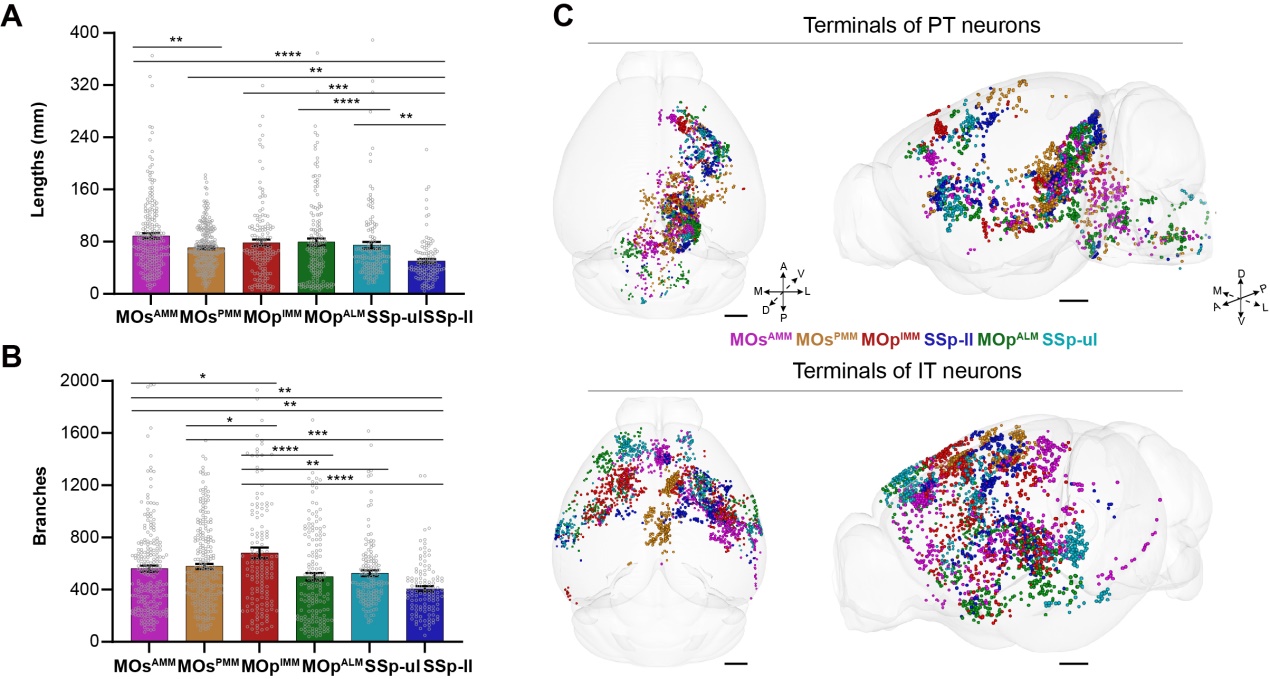


**Supplementary Fig. 7 | Morphological characteristics of reconstructed PT and IT neurons.** A, Quantification of total lengths. B, Comparison of total branches. One-way ANOVA followed by Tukey’s post hoc tests, **p* < 0.05, ***p* < 0.01, ****p* < 0.001, *****p* < 0.0001. C, Whole-brain distribution of axonal terminals of reconstructed PT and IT neurons. Dots represent axonal terminals. Colors indicate different source subregions. Scale bars, 1 mm.


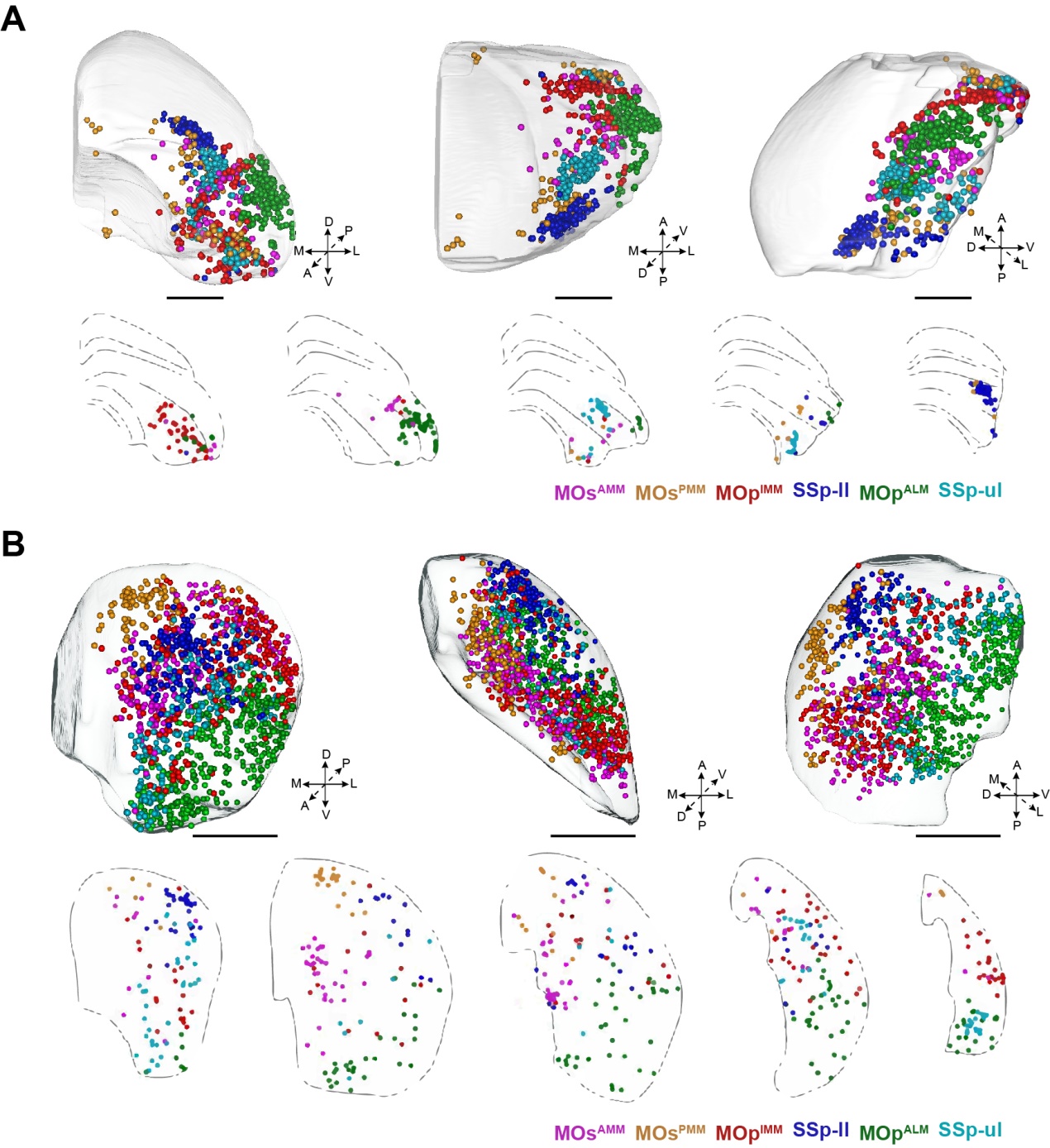


**Supplementary Fig. 8 | Axon-terminal distribution of PT and IT neurons in the SC and CP.** A, The terminal distribution of PT neurons in the SC. B, The terminal distribution of IT neurons in the CP. A-B: Scale bars, 1 mm.


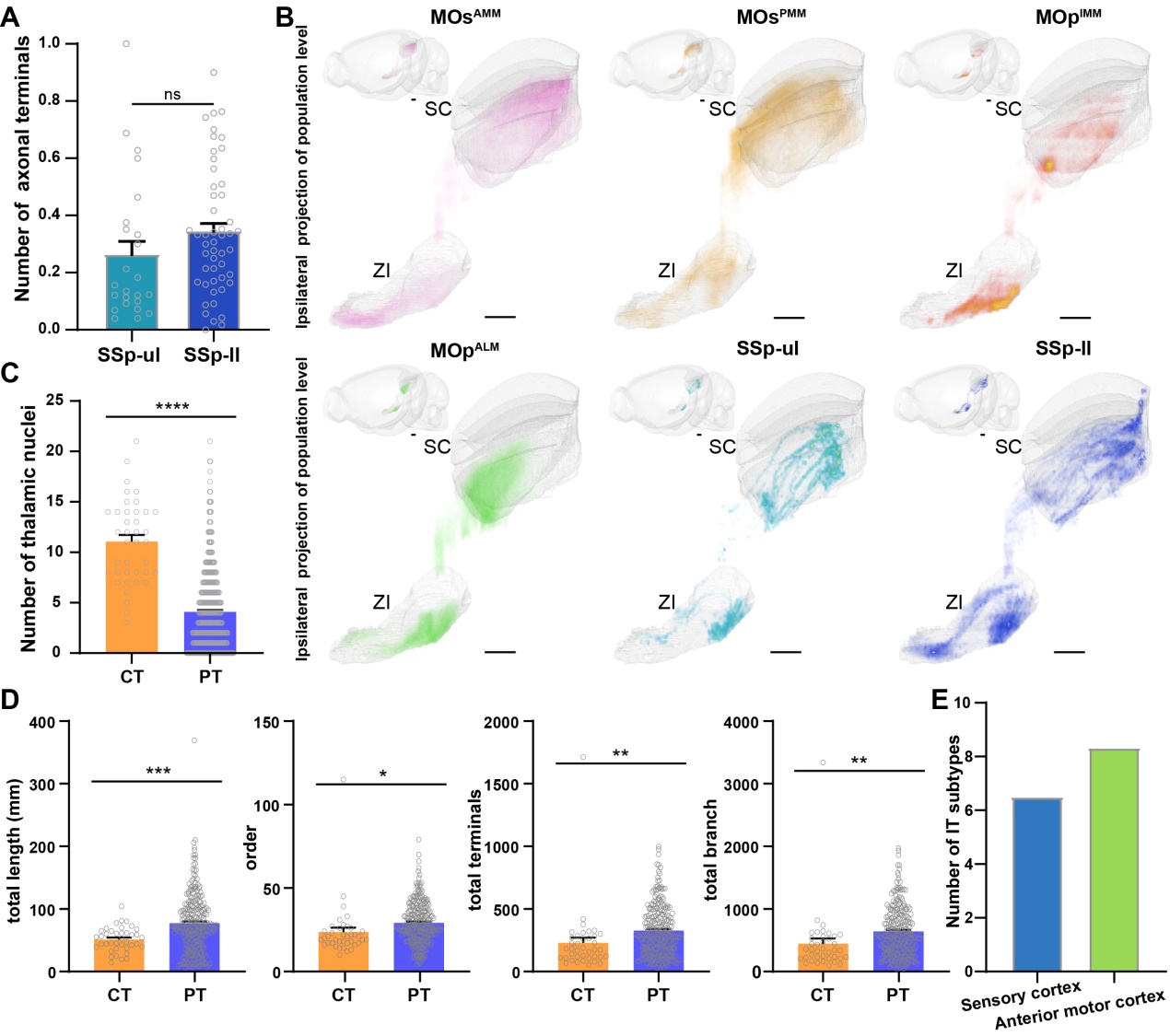


**Supplementary Fig. 9 | Projection and morphological diversity of PT neurons.** A, Distribution of the axonal terminals from PT neurons in the SSp-ul and SSp-ll. B, Distribution of axonal fibers showing population-level projection patterns of PT neurons in the ipsilateral ZI and SC. Scale bars, 1 mm. C, PT neurons target less thalamic nuclei compared with CT neurons. Each grey circle represents one neuron. D, Comparison of morphological characteristics of PT and CT neurons. E, Number of subtypes of IT neurons in sensory cortex and anterior motor cortex. Two-tailed Student’s t-test, ‘ns’ indicates no significant difference, p > 0.05. **p* < 0.05, ***p* < 0.01, ****p* < 0.001, *****p* < 0.0001.


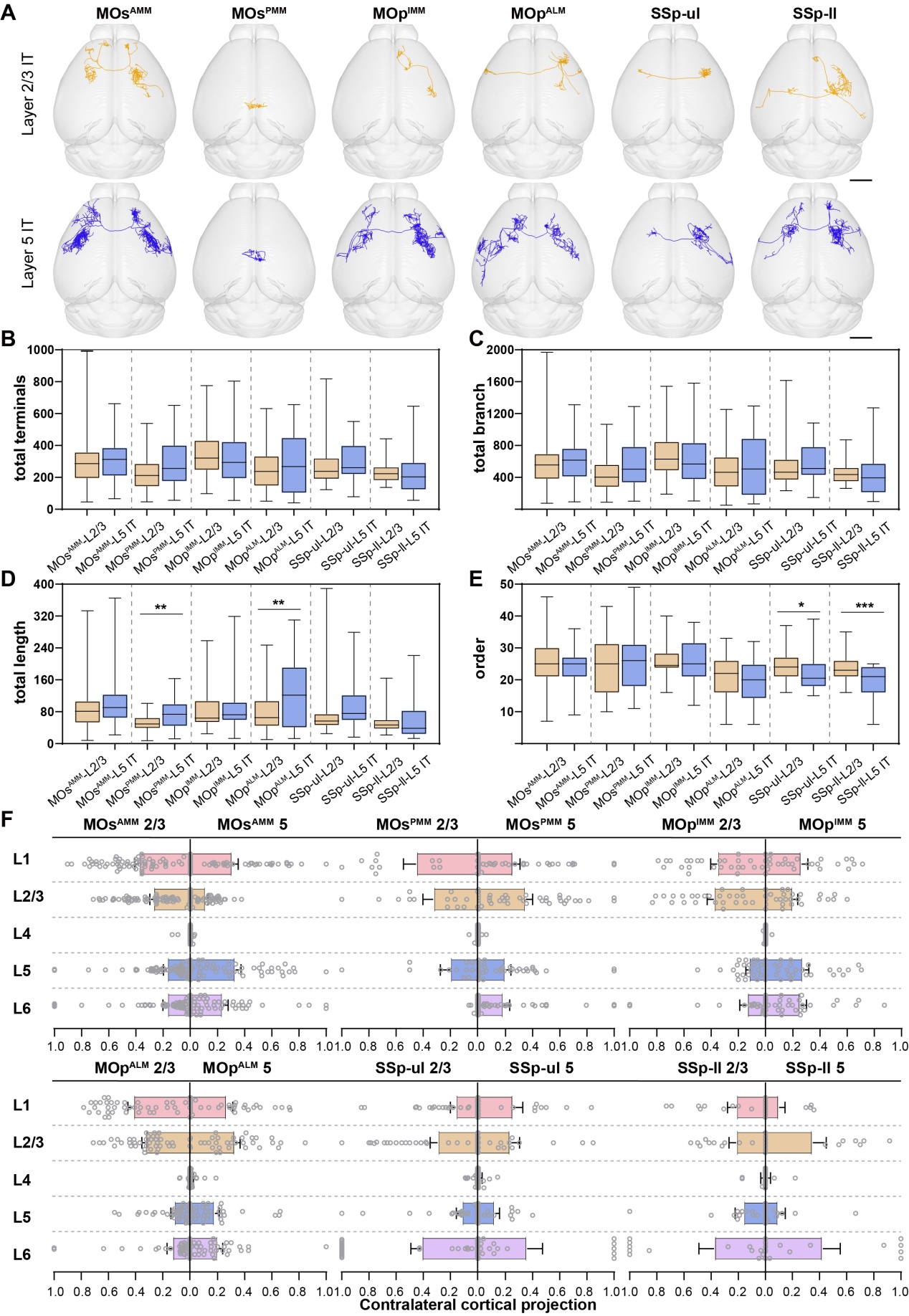


**Supplementary Fig. 10 | Comparison of projection properties between layer 2/3 and layer 5 IT neurons.** A, Representative long-range projection patterns of layer 2/3 and layer 5 IT neurons in each cortical region. Scale bars, 1 mm. B-E, Comparison of the numbers of total terminals, branches, lengths, and orders between the layer 2/3 and layer 5 IT neurons. Two-tailed Student’s t-test, **p* < 0.05, ***p* < 0.01, ****p* < 0.001, *****p* < 0.0001. F, Layer 2/3 and layer 5 IT neurons differentially innervate different layers of the contralateral cortex. Each circle represents a single IT neuron. Error bars, ± s.e.m.


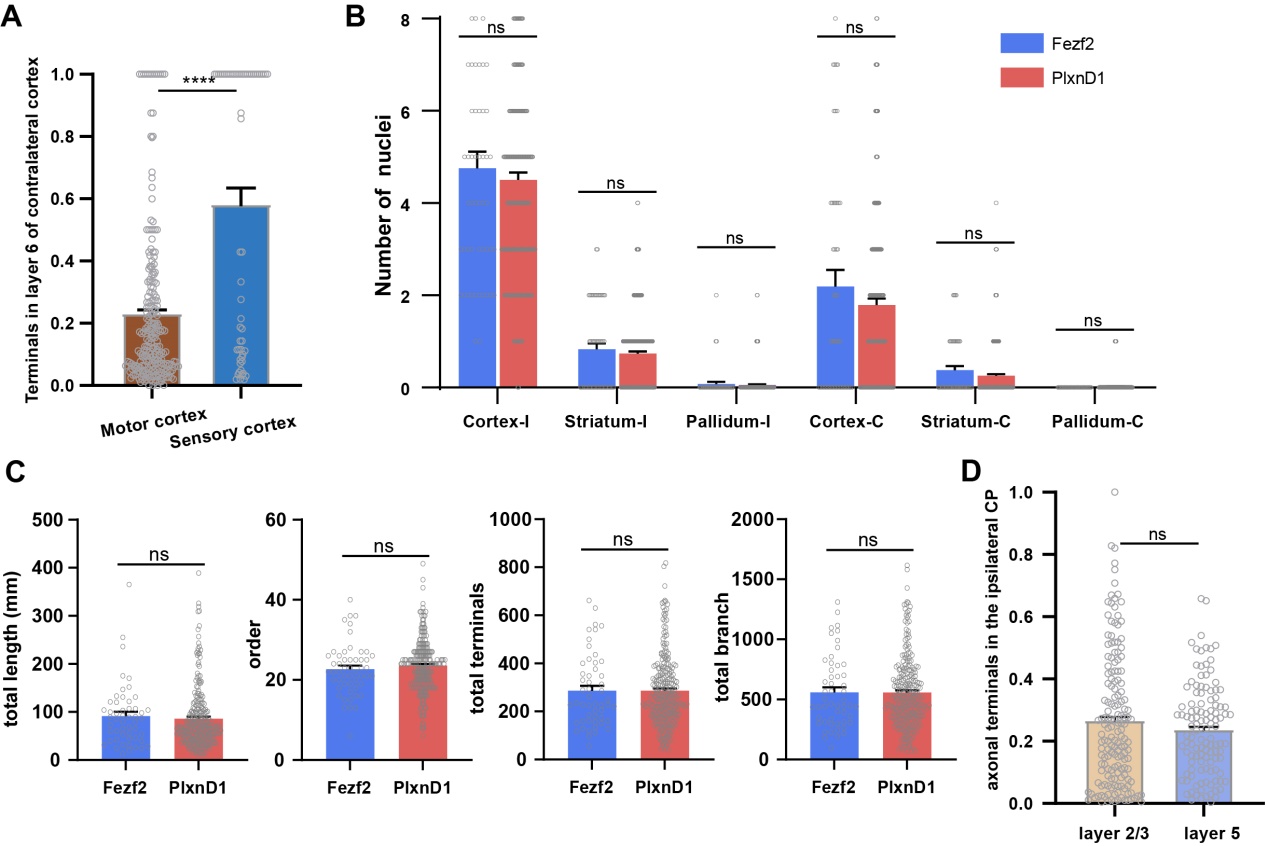


**Supplementary Fig. 11 | Detailed comparison of IT neurons labeled by *Fezf2* and *PlxnD1* driver mice.** A, IT neurons in layer 2/3 and layer 5 of the SSp-ul and SSp-ll had more axonal terminals in layer 6 of the contralateral cortex compared to those of the motor cortex. B, *Fezf2+* and *PlxnD1+* IT neurons share similar numbers of projection targets. C, Morphological characteristics analysis. D, The number of axonal terminals in layer 6 of the contralateral cortex from IT neurons in layers 2/3 and 5 of the sensory and motor cortices. Two-tailed Student’s t-test. ‘ns’ indicates no significant difference, *p* > 0.05. *****p* < 0.0001.


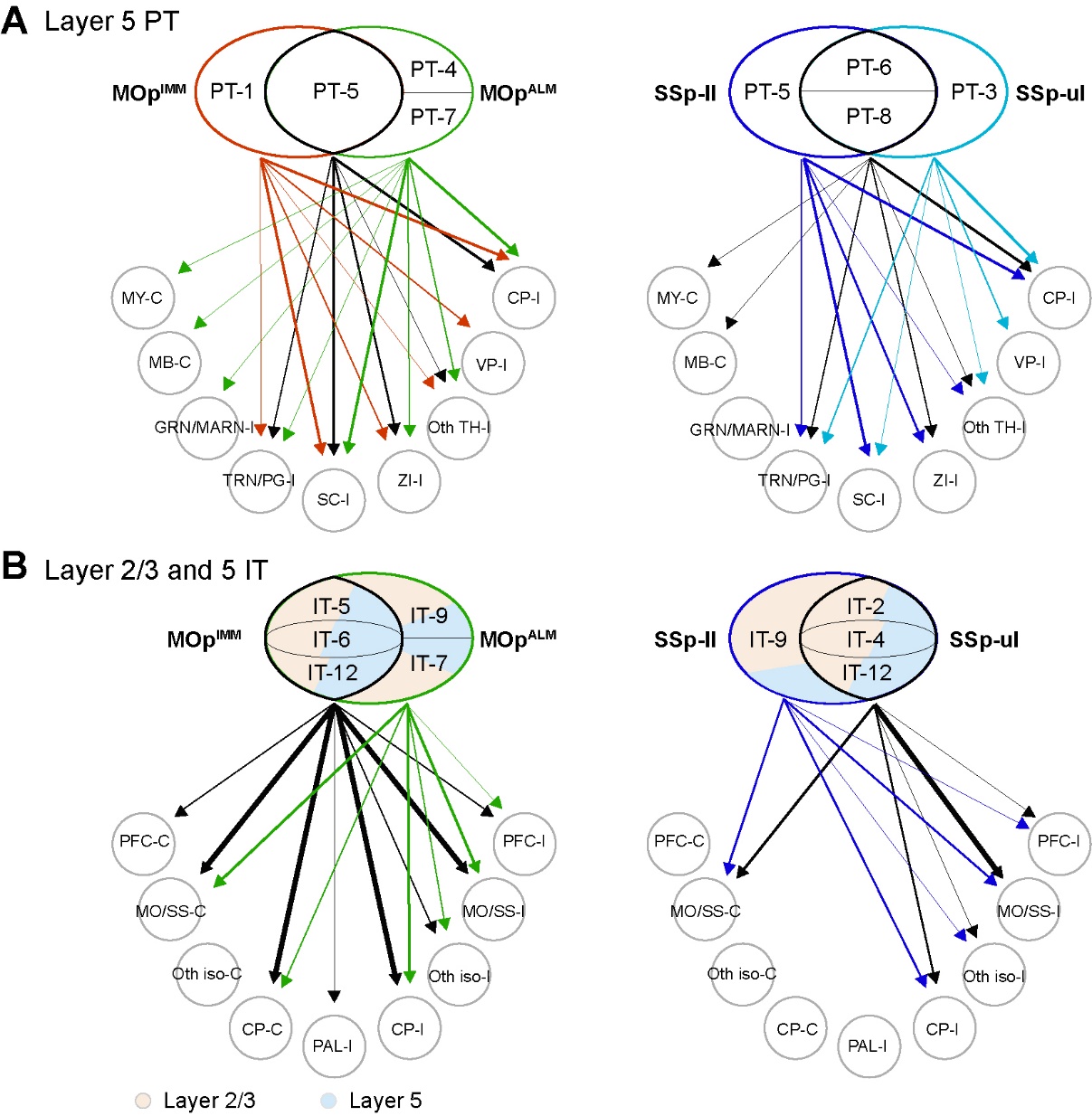


**Supplementary Fig. 12 | Long-range projection of cortical PT and IT neurons in the primary motor cortex and primary somatosensory cortex at the single-cell level.** A, Comparison of long-range projections between PT neurons in the primary motor cortex and those in primary somatosensory cortex. Line thickness indicates connection strength. B, Comparing long-range projections of IT neurons in layer 2/3 and layer 5 from the primary motor cortex and those from the primary somatosensory cortex. The abbreviations of brain regions are provided in Supplementary Table 1.


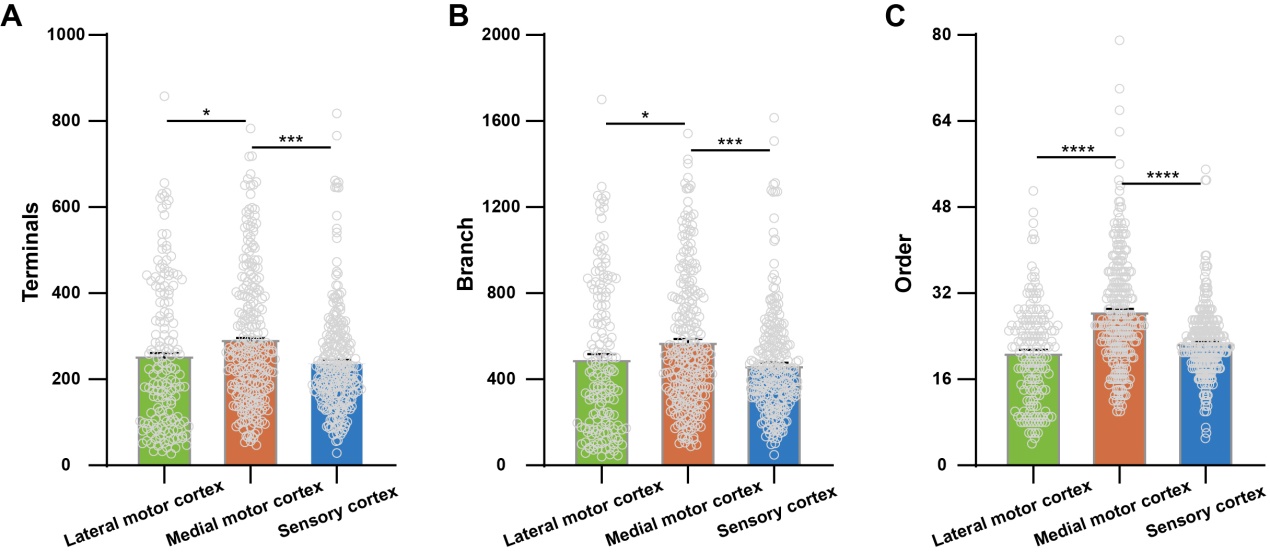


**Supplementary Fig. 13 | The number of axonal terminals, branches, and orders of axonal branches from pyramidal neurons in the sensory cortex, lateral motor cortex, and medial motor cortex.** One-way ANOVA followed by Tukey’s post hoc tests, **p* < 0.05, ****p* < 0.001, *****p* < 0.0001.

**Supplementary Table. 1| The abbreviation of brain regions.**

| Abbreviation | Full name |
| --- | --- |
| ACA  AD  AI  AM  APN  AUD  AV  CFA  CL  CM  CN  CP  Cpd  CS  CTXsp  CU  ECT  Eth  FF  FRP  GP  GPe  GPi  GRN  HIP  HY  IAD  IAM  IC  ILA  IMD  IO  IRN  LD  LDT  LP  MARN  MB  MD  MDRN  MOp  MOp^ALM^  MOp^IMM^  MOs  MOs^AMM^  MOs^PMM^  MRN  MV  MY  NDB  NPC  OLF  ORB  P  PAG  PAL  PARN  PB  PCG  PCN  PERI  PF  PG  PH  PL  PO  PoT  PPN  PRNc  PRNr  PSV  PTLp  PVT  Py  RE  RFA  RH  RN  RSP  RT  SC  SI  SNr  SPVC  SPVI  SPVO  SSp  SSp-ll  SSp-ul  SSs  STN  STR  SUB  SUV  TEa  TH  TRN  VAL  VIS  VM  VP  VPL  VPM  VTA  XII  ZI | Anterior cingulate area  Anterodorsal nucleus  Agranular insular area  Anteromedial nucleus  Anterior pretectal nucleus  Auditory areas  Anteroventral nucleus of thalamus  Caudal forelimb area  Central lateral nucleus of the thalamus  Central medial nucleus of the thalamus  Cochlear nuclei  Caudoputamen  Cerebal peduncle  Superior central nucleus raphe  Cortical subplate  Cuneate nucleus  Ectorhinal area  Ethmoid nucleus of the thalamus  Fields of Forel  Frontal pole, cerebral cortex  Globus pallidus  Globus pallidus, external segment  Globus pallidus, internal segment  Gigantocellular reticular nucleus  Hippocampal formation  Hypothalamus  Interanterodorsal nucleus of the thalamus  Interanteromedial nucleus of the thalamus  Inferior colliculus  Infralimbic area  Intermediodorsal nucleus of the thalamus  Inferior olivary complex  Intermediate reticular nucleus  Lateral dorsal nucleus of thalamus  Laterodorsal tegmental nucleus  Lateral posterior nucleus of the thalamus  Magnocellular reticular nucleus  Midbrain  Mediodorsal nucleus of thalamus  Medullary reticular nucleus  Primary motor area  Anterior-lateral motor  Intermediate-medial motor area  Secondary motor area  Anterior-medial motor area  Posterior-medial motor area  Midbrain reticular nucleus  Medial vestibular nucleus  Medulla  Diagonal band nucleus  Nucleus of the posterior commissure  Olfactory areas  Orbital area  Pons  Periaqueductal gray  Pallidum  Parvicellular reticular nucleus  Parabrachial nucleus  Pontine central gray  Paracentral nucleus  Perirhinal area  Parafascicular nucleus  Pontine gray  Posterior hypothalamic nucleus  Prelimbic area  Posterior complex of the thalamus  Posterior triangular thalamic nucleus  Pedunculopontine nucleus  Pontine reticular nucleus, caudal part  Pontine reticular nucleus  Principal sensory nucleus of the trigeminal  Posterior parietal association areas  Paraventricular nucleus of the thalamus  Pyramid  Nucleus of reuniens  Rostral forelimb area  Rhomboid nucleus  Red nucleus  Retrosplenial area  Reticular nucleus of the thalamus  Superior colliculus  Substantia innominata  Substantia nigra, reticular part  Spinal nucleus of the trigeminal, caudal part  Spinal nucleus of the trigeminal, interpolar part  Spinal nucleus of the trigeminal, oral part  Primary somatosensory area  Primary somatosensory area, lower limb  Primary somatosensory area, upper limb  Supplemental somatosensory area  Subthalamic nucleus  Striatum  Subiculum  Superior vestibular nucleus  Temporal association areas  Thalamus  Tegmental reticular nucleus  Ventral anterior-lateral complex of the thalamus  Visual areas  Ventral medial nucleus of the thalamus  Ventral posterior complex of the thalamus  Ventral posterolateral nucleus of the thalamus  Ventral posteromedial nucleus of the thalamus  Ventral tegmental area  Hypoglossal nucleus  Zona incerta |

**Supplementary Table. 2| Number of PT neurons of different subtypes located in different subregions.**

| **Regions**  **Subtypes** | **MOs^AMM^** | **MOs^PMM^** | **MOp^IMM^** | **MOp^ALM^** | **SSp-ul** | **SSp-ll** |
| --- | --- | --- | --- | --- | --- | --- |
| **PT-1** | 5 | 35 | 20 | 8 | 1 | 3 |
| **PT-2** | 2 | 10 | 4 | 1 | 2 | 7 |
| **PT-3** | 5 | 6 | 4 | 0 | 7 | 6 |
| **PT-4** | 14 | 24 | 9 | 19 | 0 | 0 |
| **PT-5** | 10 | 47 | 28 | 21 | 4 | 12 |
| **PT-6** | 9 | 26 | 5 | 7 | 6 | 8 |
| **PT-7** | 0 | 4 | 3 | 13 | 2 | 6 |
| **PT-8** | 12 | 13 | 2 | 4 | 5 | 9 |
| **Total** | 57 | 165 | 75 | 73 | 27 | 51 |

**Supplementary Table. 3| Number of IT neurons of different subtypes located in different subregions.**

| **Regions**  **Subtypes** | **MOs^AMM^** | **MOs^PMM^** | **MOp^IMM^** | **MOp^ALM^** | **SSp-ul** | **SSp-ll** |
| --- | --- | --- | --- | --- | --- | --- |
| **IT-1** | 15 | 3 | 3 | 2 | 2 | 1 |
| **IT-2** | 0 | 3 | 4 | 2 | 18 | 12 |
| **IT-3** | 9 | 6 | 1 | 5 | 8 | 4 |
| **IT-4** | 0 | 9 | 3 | 1 | 30 | 17 |
| **IT-5** | 13 | 0 | 9 | 10 | 1 | 0 |
| **IT-6** | 32 | 0 | 15 | 21 | 1 | 1 |
| **IT-7** | 11 | 1 | 3 | 9 | 4 | 2 |
| **IT-8** | 21 | 3 | 5 | 2 | 7 | 1 |
| **IT-9** | 13 | 5 | 8 | 13 | 11 | 11 |
| **IT-10** | 9 | 8 | 3 | 7 | 2 | 0 |
| **IT-11** | 5 | 37 | 1 | 0 | 5 | 3 |
| **IT-12** | 2 | 4 | 11 | 10 | 25 | 13 |
| **Total** | 130 | 79 | 66 | 82 | 114 | 65 |

**Materials and methods**

**Animals**

We crossed 2~6 months old *PlxnD1-2A-CreER* and *Fezf2-2A-CreER* mice[21, 30] (a gift from Josh Huang’s laboratory, Cold Spring Harbor) with *Rosa26-loxp-stop-loxp-flpo (LSL-Flp)* mouse (The Jackson Laboratory stock 028584) to use AAV to specifically and sparsely label IT and PT neurons in the sensorimotor cortex. In addition, the *PlxnD1-2A-CreER* and *Fezf2-2A-CreER* mice were crossed with Rosa26-Tcf/Lef-LSL-H2B-GFP mice (The Jackson Laboratory stock 032577) to determine the whole-brain distribution of *PlxnD1+* and *Fezf2+* neurons. All mice were placed in an SPF-grade animal house with adequate access to water and food, constant temperature and humidity, and were given 12 hours of light daily. All experiments and analyses were conducted without gender distinction. All experiments *involving* animals were conducted in accordance with the requirements of Hubei Provincial Laboratory Animal Management Committee and the Animal Ethics of Huazhong University of Science and Technology.

**Stereotactic injection of virus**

An appropriate amount of anesthetics (1% pentobarbital sodium in 0.9% saline) was intraperitoneally injected into mice (0.1 mL anesthetic per 10g bodyweight, 0.1 mL/10 g). After deep anesthesia, mice were placed on an adapter and fixed on a stereo locator. The scalp was cut open with anatomical scissors to expose the skull. Subsequently, the prepared microinjection needle was fixed on the stereo locator and the cold light lamp was turned on to irradiate the skull. We moved the manipulator arm of the stereo locator until the tip reached the bregma and then set the position zero as a reference. Based on Z-axis reading, the medial and lateral axis and the anterior and posterior axis of the skull were adjusted to level respectively. Based on the three-dimensional coordinates of the target brain region, we used a skull drill to slowly drill into this region. An automated syringe pump was used to withdraw an appropriate amount of virus into a microinjection needle. We then moved the needle to the target location and injected the virus.

*PlxnD1-2A-CreER* and *Fezf2-2A-CreER* mice were crossed with the *LSL-Flp* converter mouse line such that after tamoxifen (Sigma, T5648) induction, CreER-expressing neurons are converted to have constitutive Flp expression for anterograde tracing with a Flp-dependent AAV vector (pAAV-EF1a-fDIO-TVA-GFP virus (8 × 10^12^ gc/ml, UNC Vector Core (Chapel Hill)). The coordinates of the six injection sites in the sensorimotor cortex were as follows: MOs^AMM^ (AP: +1.5, ML: -0.6), MOs^PMM^ (AP: -1.5, ML: -0.75), MOp^IMM^ (AP: +0.37, ML: -1.1), MOp^ALM^ (AP: +1.5, ML: -2), SSp-ul (AP: -0.01, ML: 2.46), and SSp-ll (AP: -0.79, ML: -1.75). Where ‘A’ and ‘P’ indicate anterior and posterior directions, respectively. ‘M’ and ‘L’ represent medial and lateral respectively.

**Resin Embedding**

After the heart of anesthetized mice was perfused with 0.01M PBS (Sigma-Aldrich Inc., St Louis, MO, USA) and 4% paraformaldehyde (PFA, Sigma-Aldrich Inc., St Louis, MO, USA) of anesthetized mice, then the brain was extracted. Mouse brains were embedded with HM20 resin (Lowicryl HM20)[57]. After PFA postfixation, the brain sample was rinsed with PBS solution three times, including 2 hours each for the first two times and 12 hours for the third time. The brain sample was then dehydrated with gradient ethanol, including 75% and 95% ethanol for 2 hours respectively, and 100% ethanol three times each lasting for 2 hours. Once the dehydration procedure was completed, the samples were immersed in 50% ethanol removal solutions (xylene and anhydrous ethanol were prepared at a volume ratio of 1:1) for approximately 2 hours. The samples were then immersed in 100% xylene solutions to replace the ethanol in the tissues, which consisted consisting of three stages: 2 hours, 12 hours, and 2 hours for each stage. The permeation process involved immersing the sample in gradient preosmosis solution (resin and xylene were prepared according to the volume ratio), including 50% preosmosis solution for 2 hours, 75% for 2 hours, and 100% for three times 2 hours each. This was followed by soaking in CR-quench solutions (6 uL acetic acid /1 mL resin) for approximately 14 hours. All procedures were carried out at 4℃ in the dark. Subsequently, the mouse brain was placed in the capsule with CR-quench solution added, followed by being placed in the oven for gradient heating and polymerization. There were three steps for the polymerization process: 37℃ for 12 hours, 45℃ for 8 hours and 50℃ for 3 hours.

**Imaging**

For HM20 resin-embedded samples, fMOST system[57, 58] was used to obtain a continuous three-dimensional output and single-neuron morphological dataset of the whole brain. The embedded sample was fixed on a metal base following by the application of Na_2_CO_3_ solution. Under the action of alkaline buffer solutions, the surface fluorescent protein molecules undergo a chemical transformation and become fluorescent states. If it is necessary to obtain the cytoarchitectural information of the sample, the appropriate PI solution is added. By moving the stage, the fMOST system imaged the surface of the sample in a line scan manner, and then the diamond knife cut away the imaged surface. This allows the sample to be cut on the side. The process was repeated until the mouse brain was completely imaged. Finally, a complete and continuous whole-brain dataset with a voxel resolution of 0.35 × 0.35 × 1 μm^3^ was obtained.

**Reconstruction and visualization**

For the morphological reconstruction of a single neuron, we first converted the collected high-resolution dataset into Tdat format, imported it into Amira, applied the filament editor module to trace the neuron morphology, and saved the result in SWC format. To ensure the accuracy of the tracing path, each reconstructed neuron was manually verified.

The reconstructed neurons were registered to the CCF based on the cytoarchitecture information, and then the dendrites and axons of the registered neurons were split and imported into the Amira software, respectively. The Identify Graphs interface was used to calculate the length, number of branches, and orders of axonal branches. The NeuroGPS software[28, 29] was used to identify and count the terminals in different brain regions.

**Statistics and analysis**

The traced neurons were examined independently by three individuals to correct for possible errors in the reconstruction process. Afterwards, the registration method developed by our research group[59] was employed, and the reconstructed neurons were registered to the Allen CCFv3 using the cell architecture channel of the images. Subsequently, the dendrites and axons of the registered neurons were separated. The length, number of branches, and number of bifurcations of dendrites and axons were calculated by using Amira software (v5.2.2, Mercury Computer Systems, San Diego, CA, United States). NeuroGPS software developed by our research group[28, 29] was utilized to identify and calculate the number of terminals of each neuron in different brain regions of the entire brain. The neurons were classified based on their downstream target areas. All reconstructed neurons were collected from 70 brain samples. We calculated the ratio of the number of axonal terminals in each downstream target region to the total number of terminals of a single neuron. Based on this ratio, we used SPSS software (v22, IBM, New York, United States) to perform unsupervised hierarchical clustering for all neurons. GraphPad Prism v.8.02 software was used to draw statistical graphs and calculate the single-factor variance. The distribution of axon terminals of IT and PT neurons was imported into GraphPad Prism v.8.02 software to draw heatmaps of projection matrix. Two-tailed Student’s t-test and one-way ANOVA followed by Tukey’s post hoc tests were performed using Graphpad Prism v.8.02.
