# Supplementary figures and images for: From Individual to Population: Circuit Organization of Pyramidal Tract and Intratelencephalic Neurons in Mouse Sensorimotor Cortex
Source: Research (Wash D C). 2024 Oct 7;7:0470. doi: 10.34133/research.0470 (PMC11456696; doi:10.34133/research.0470)

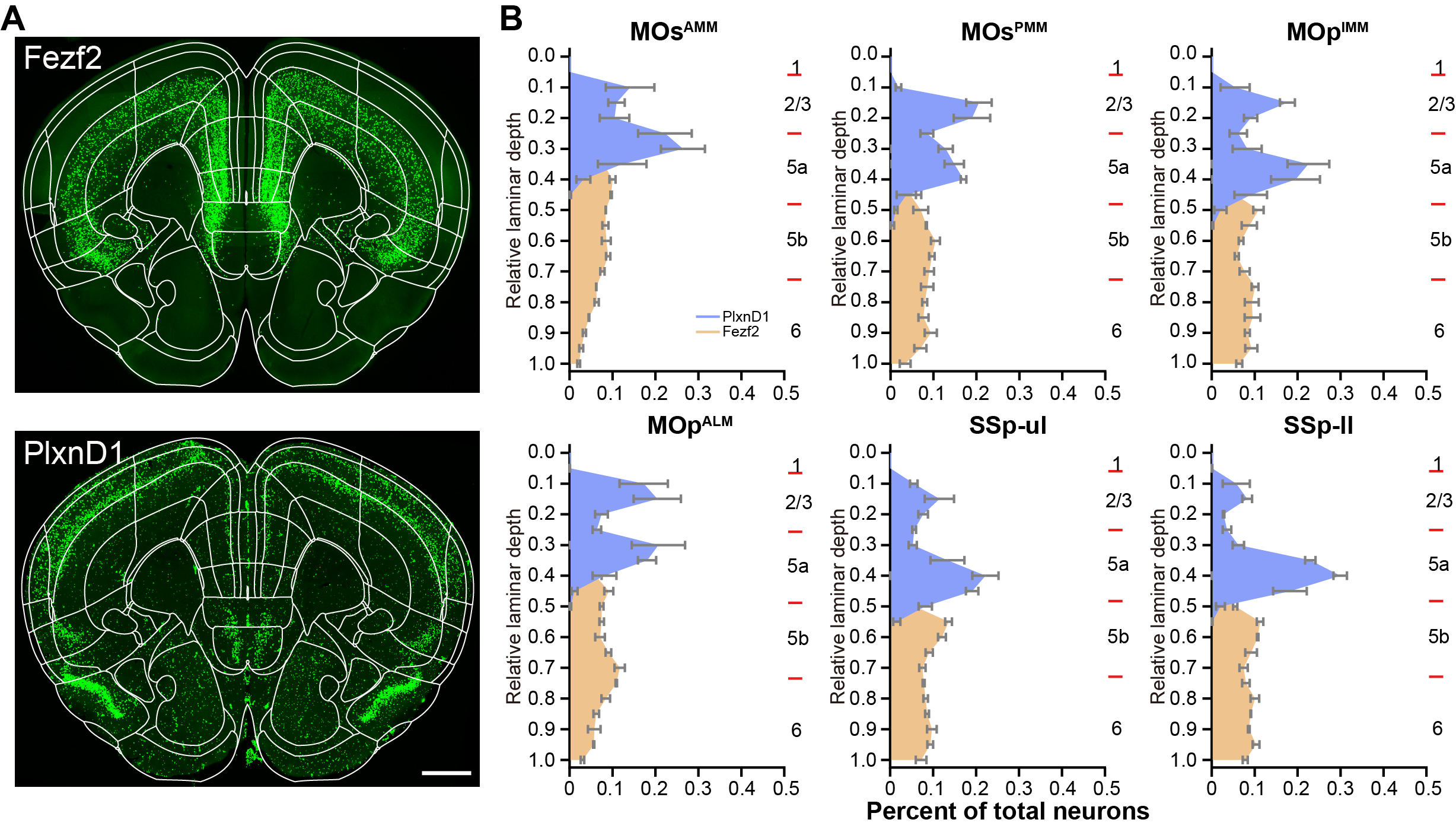

Supplement: Supplementary 1 — Figs. S1 to S13 Tables S1 to S3 Materials and Methods References [57–59] [file research.0470.f1.zip › Sup_Figure1.jpg]

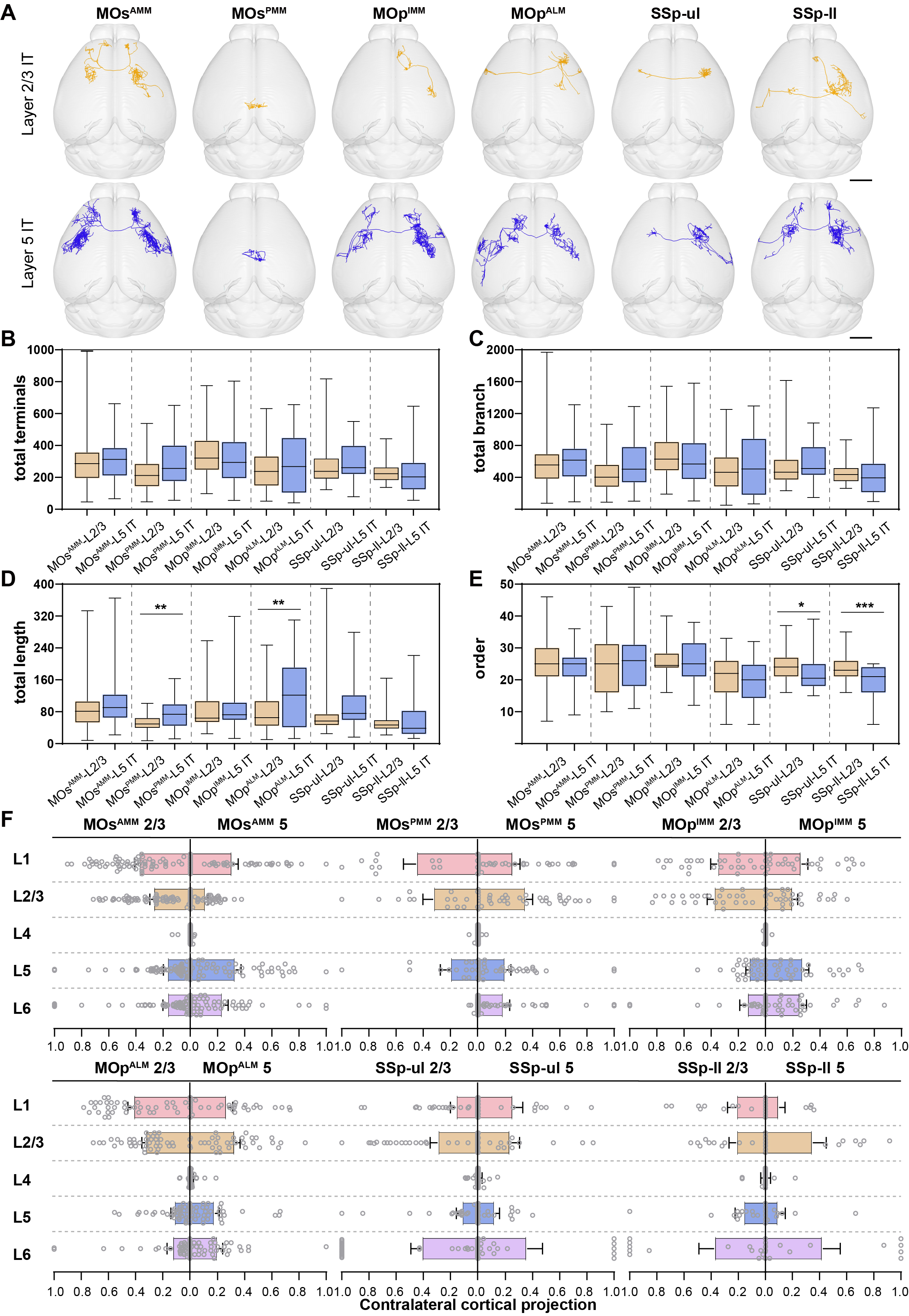

Supplement: Supplementary 1 — Figs. S1 to S13 Tables S1 to S3 Materials and Methods References [57–59] [file research.0470.f1.zip › Sup_Figure10.jpg]

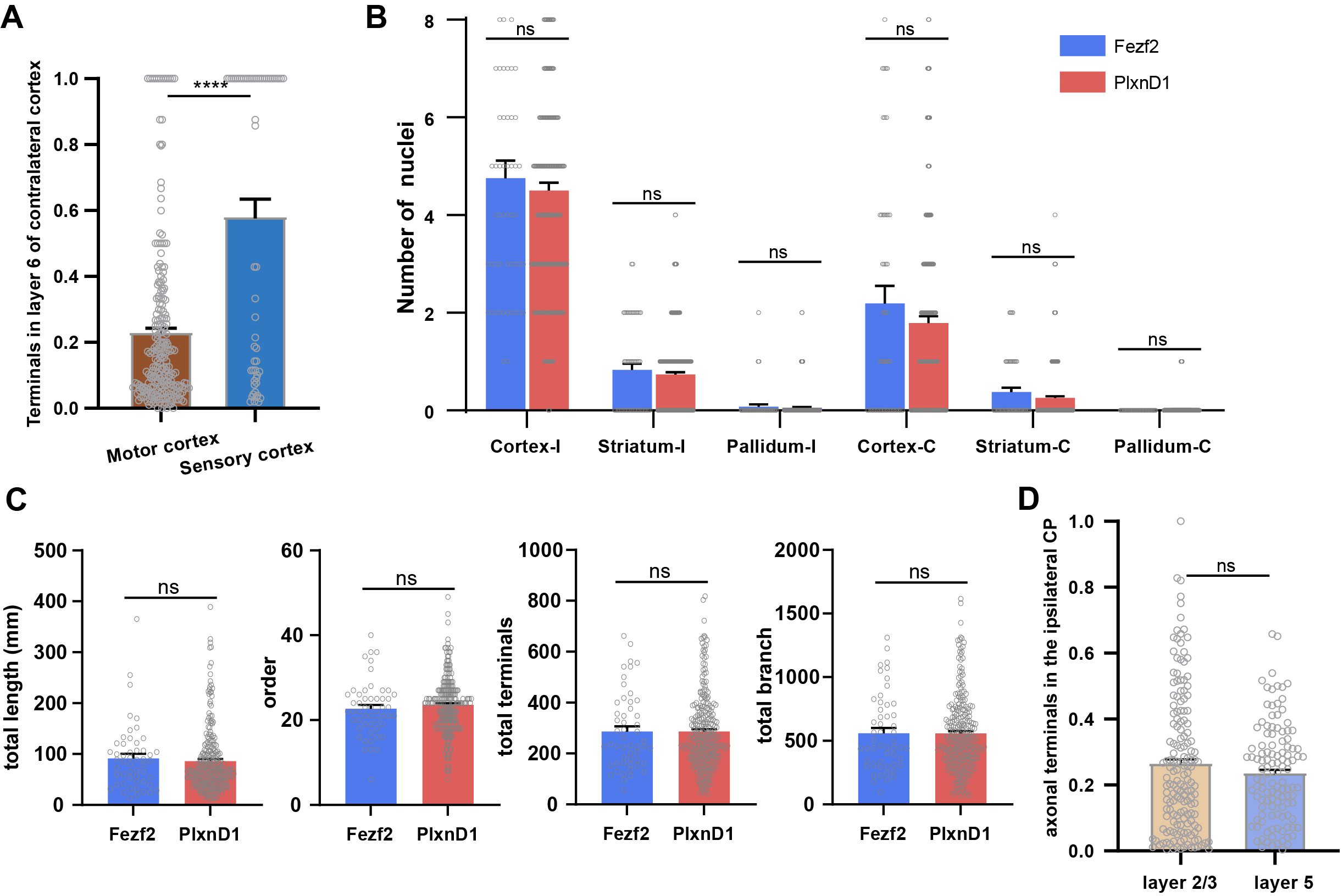

Supplement: Supplementary 1 — Figs. S1 to S13 Tables S1 to S3 Materials and Methods References [57–59] [file research.0470.f1.zip › Sup_Figure11.jpg]

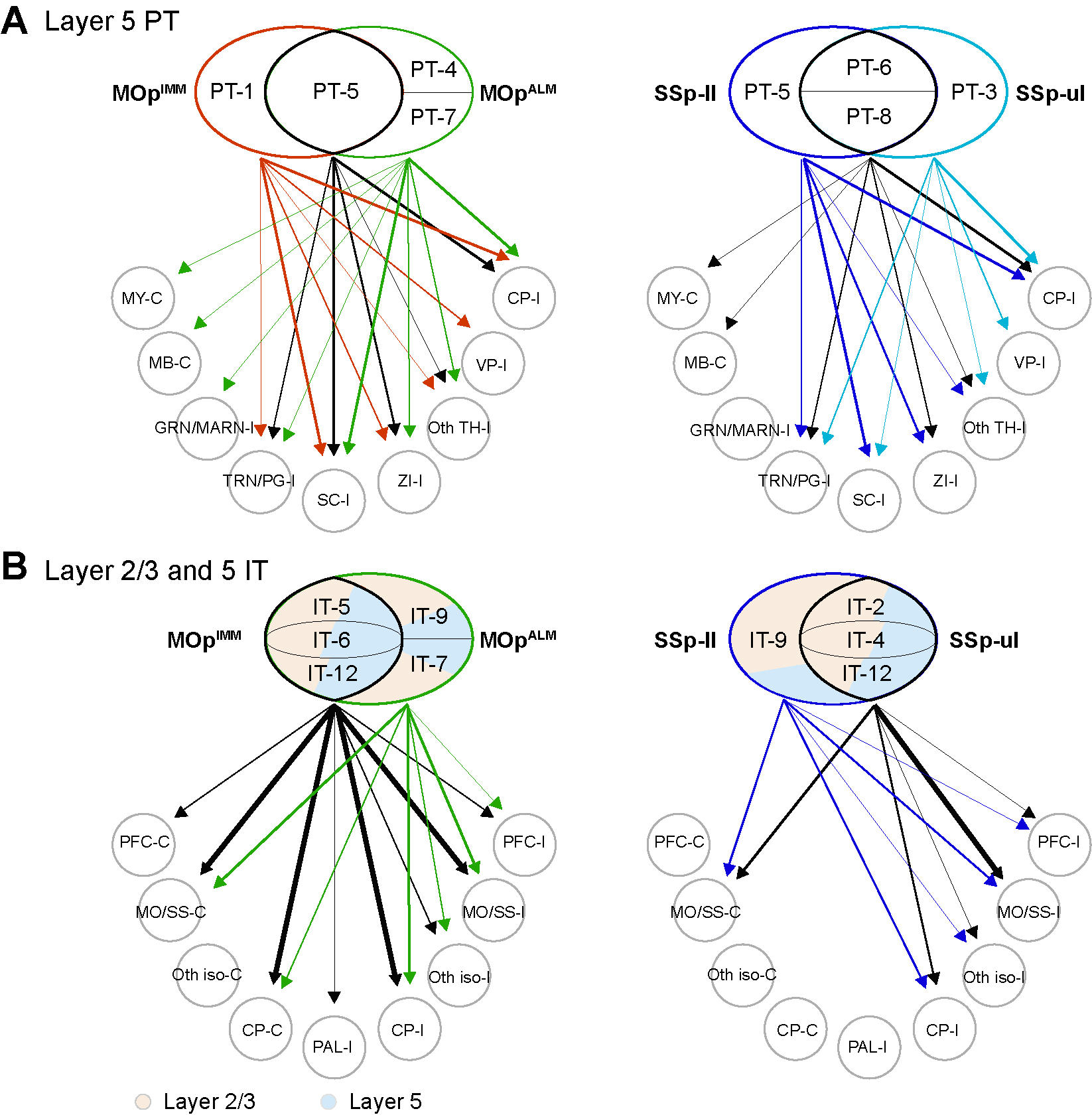

Supplement: Supplementary 1 — Figs. S1 to S13 Tables S1 to S3 Materials and Methods References [57–59] [file research.0470.f1.zip › Sup_Figure12.jpg]

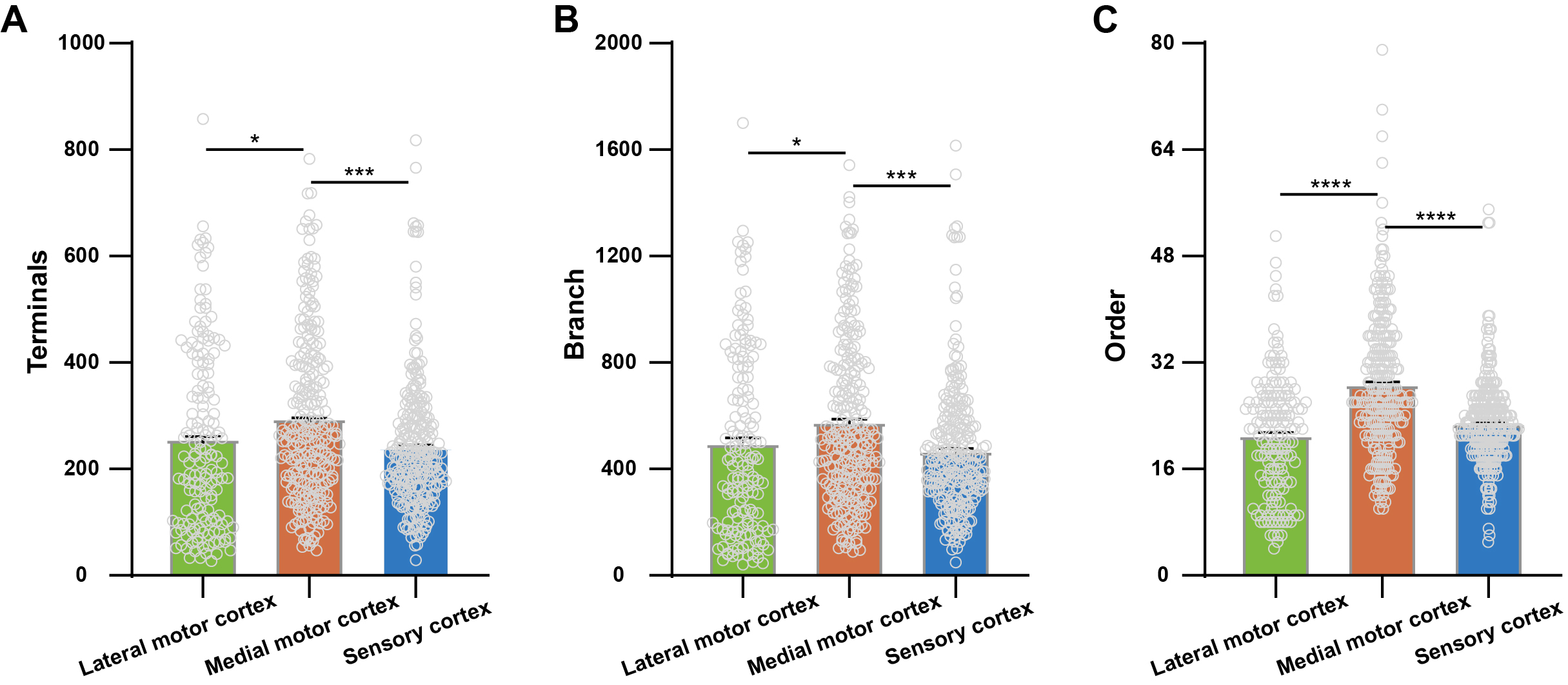

Supplement: Supplementary 1 — Figs. S1 to S13 Tables S1 to S3 Materials and Methods References [57–59] [file research.0470.f1.zip › Sup_Figure13.jpg]

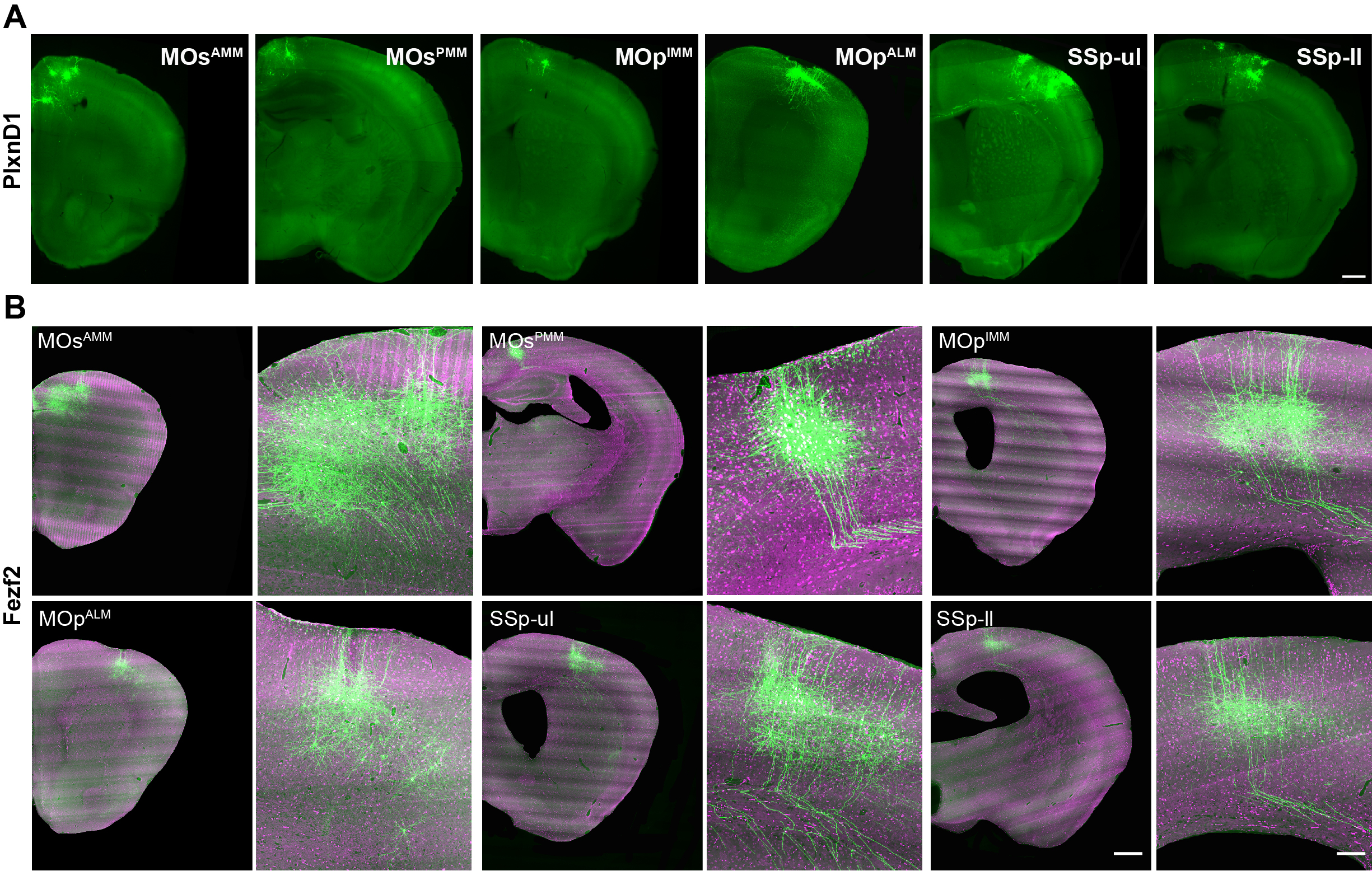

Supplement: Supplementary 1 — Figs. S1 to S13 Tables S1 to S3 Materials and Methods References [57–59] [file research.0470.f1.zip › Sup_Figure2.jpg]

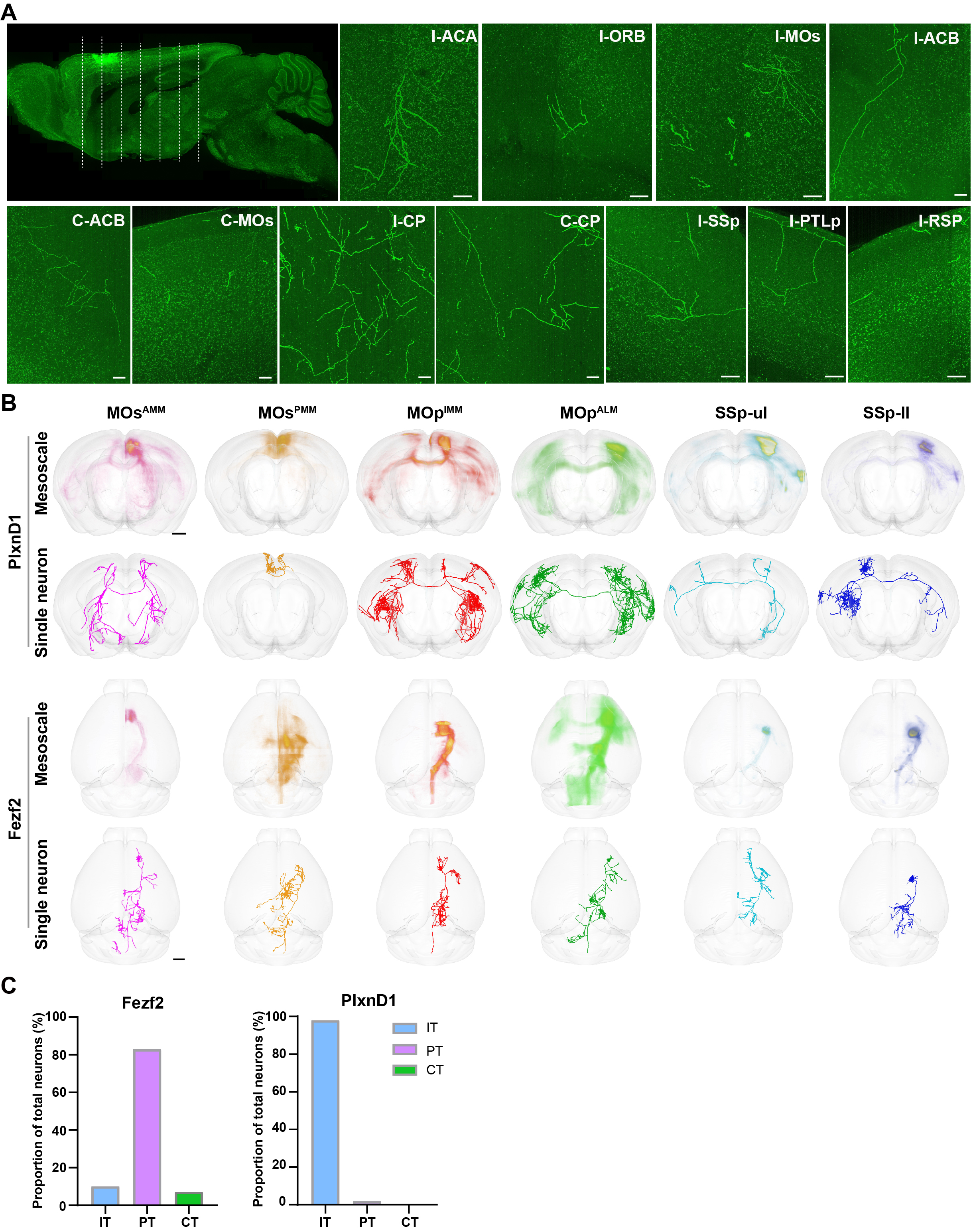

Supplement: Supplementary 1 — Figs. S1 to S13 Tables S1 to S3 Materials and Methods References [57–59] [file research.0470.f1.zip › Sup_Figure3.jpg]

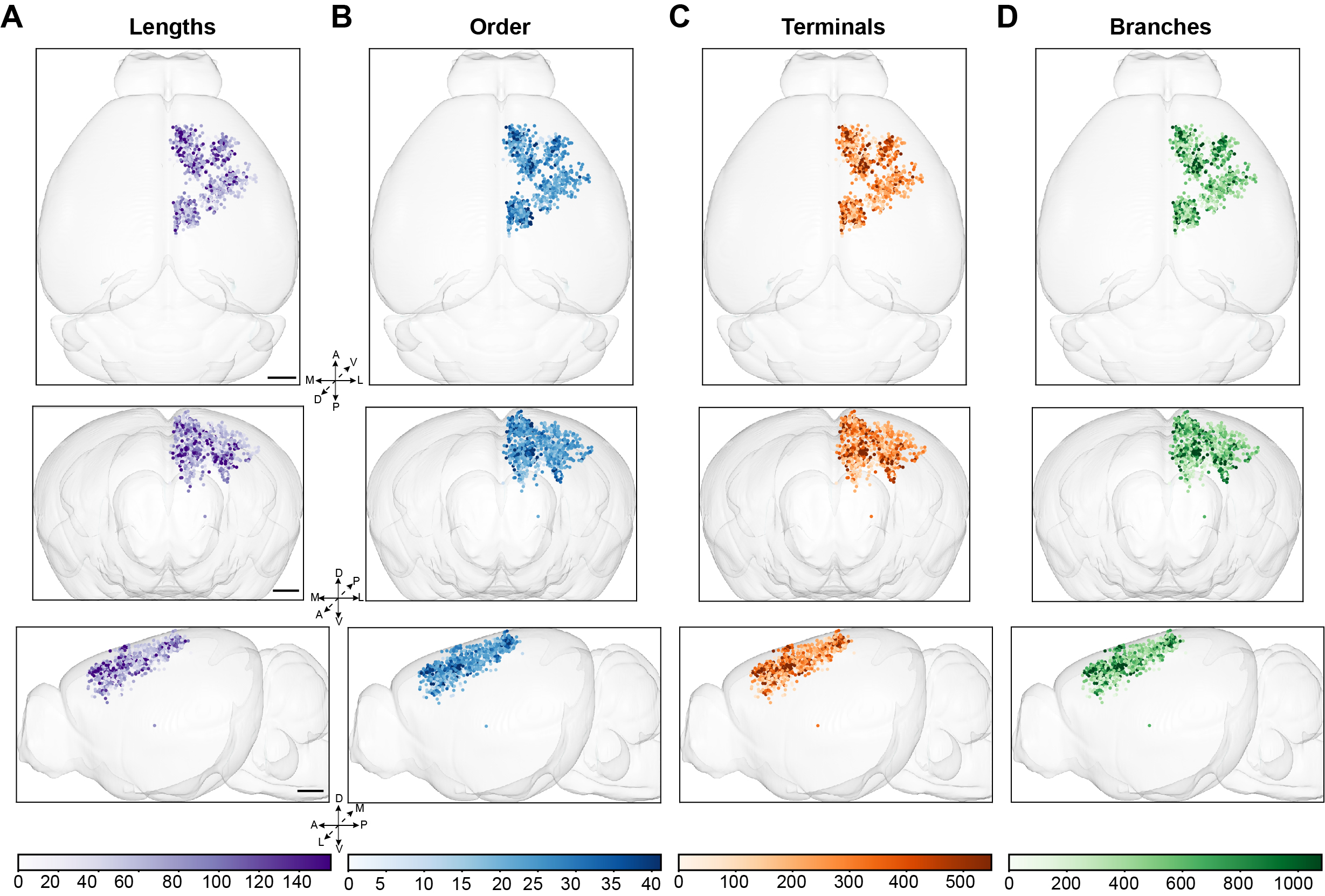

Supplement: Supplementary 1 — Figs. S1 to S13 Tables S1 to S3 Materials and Methods References [57–59] [file research.0470.f1.zip › Sup_Figure4.jpg]

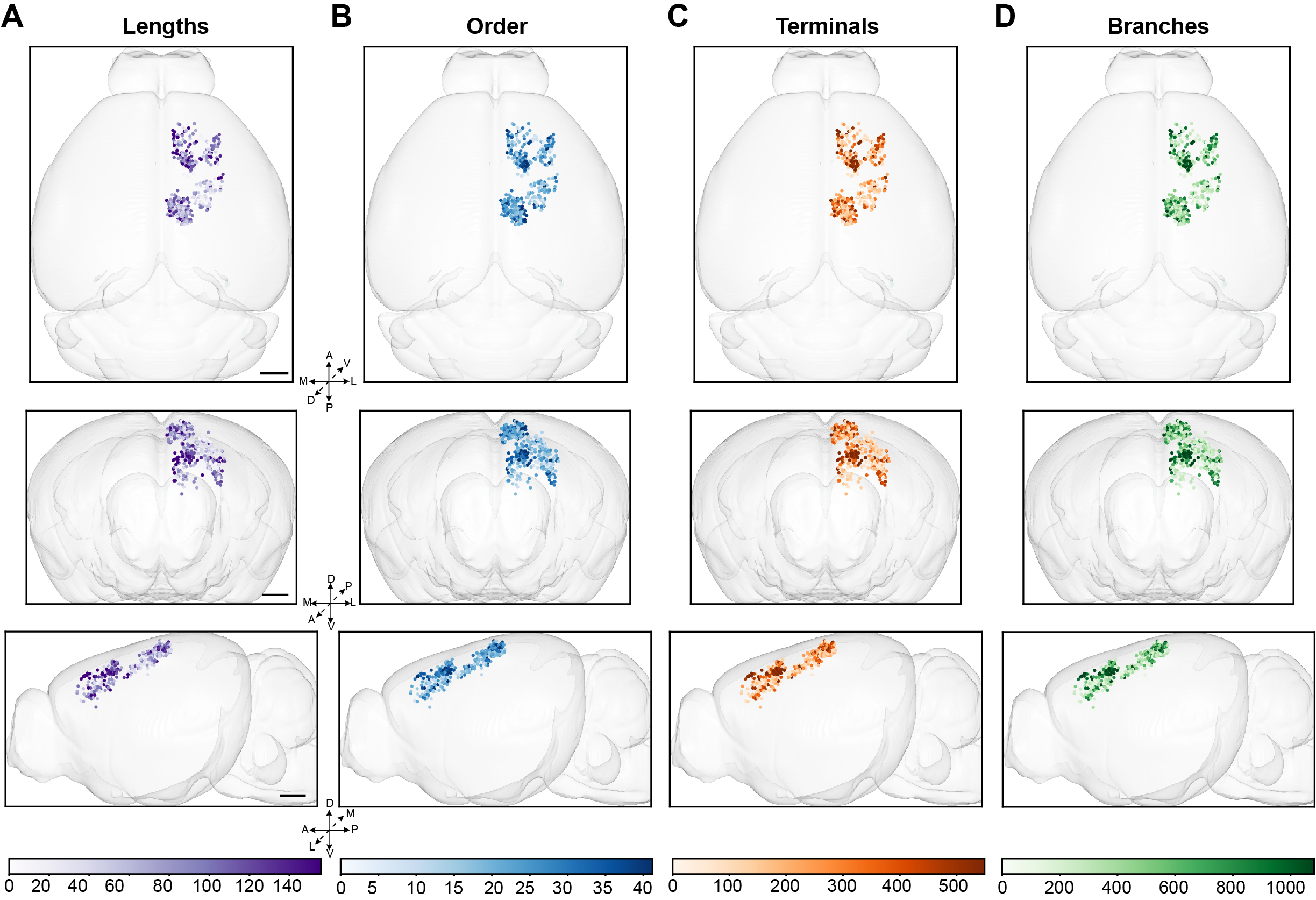

Supplement: Supplementary 1 — Figs. S1 to S13 Tables S1 to S3 Materials and Methods References [57–59] [file research.0470.f1.zip › Sup_Figure5.jpg]

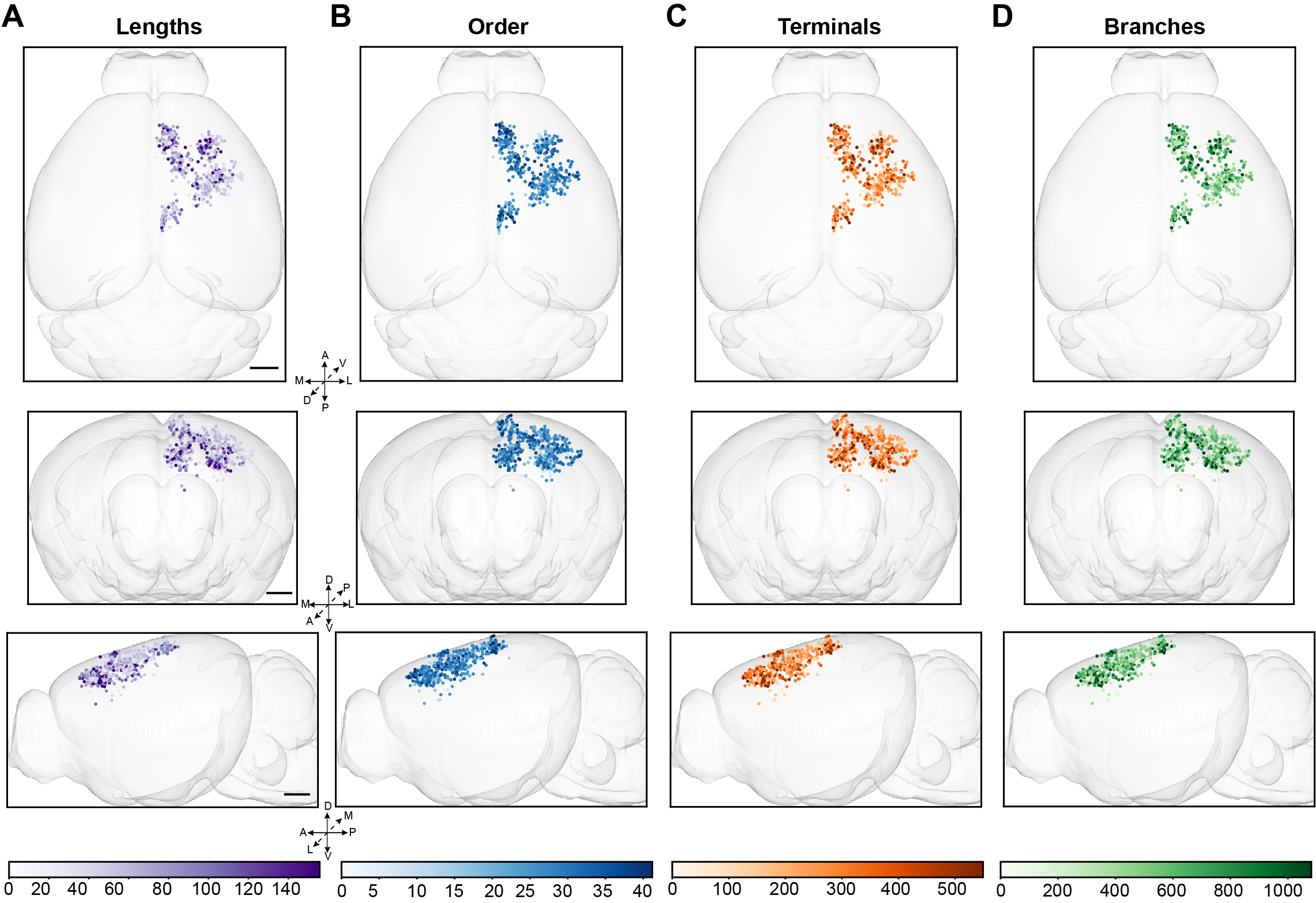

Supplement: Supplementary 1 — Figs. S1 to S13 Tables S1 to S3 Materials and Methods References [57–59] [file research.0470.f1.zip › Sup_Figure6.jpg]

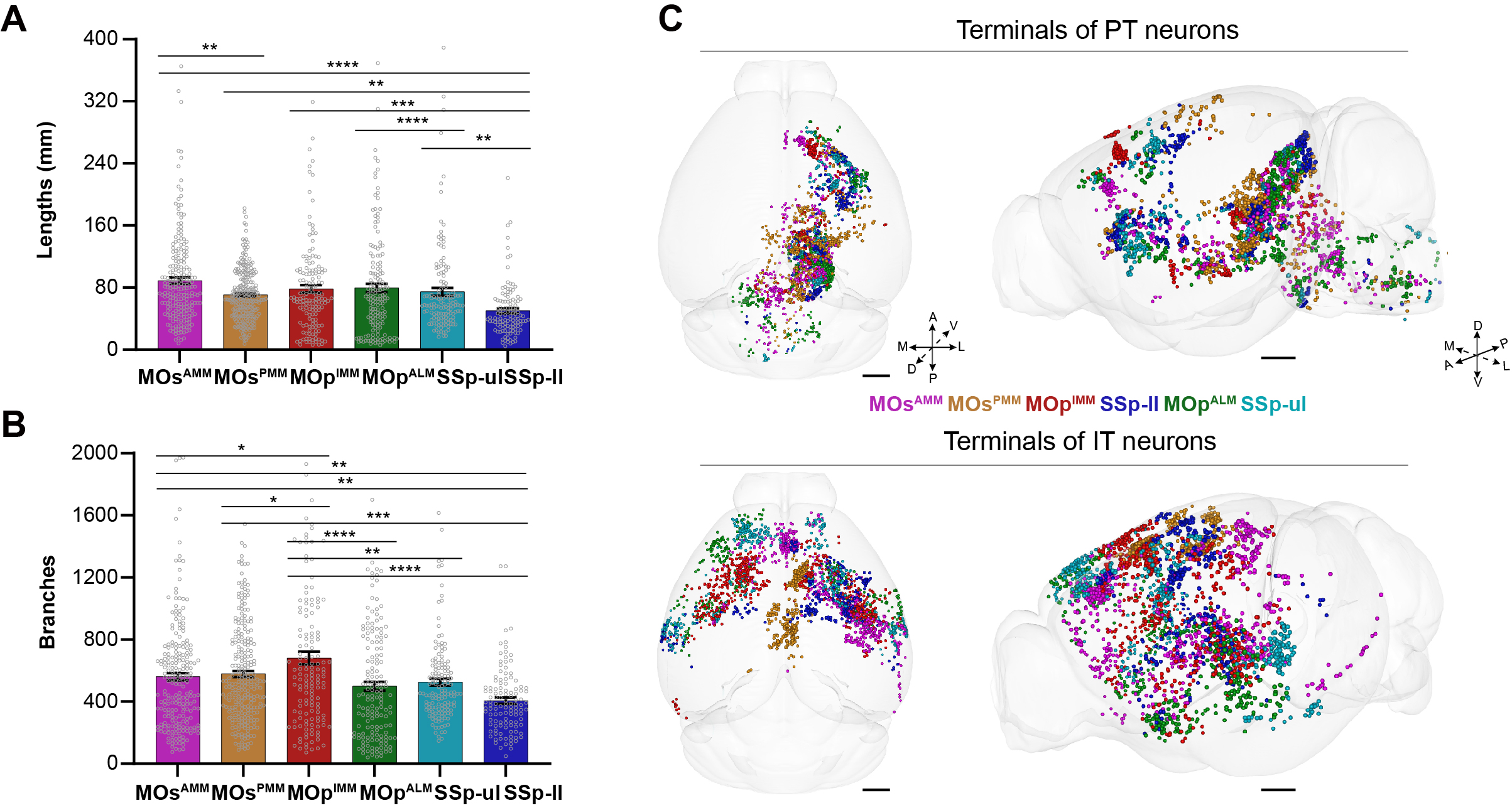

Supplement: Supplementary 1 — Figs. S1 to S13 Tables S1 to S3 Materials and Methods References [57–59] [file research.0470.f1.zip › Sup_Figure7.jpg]

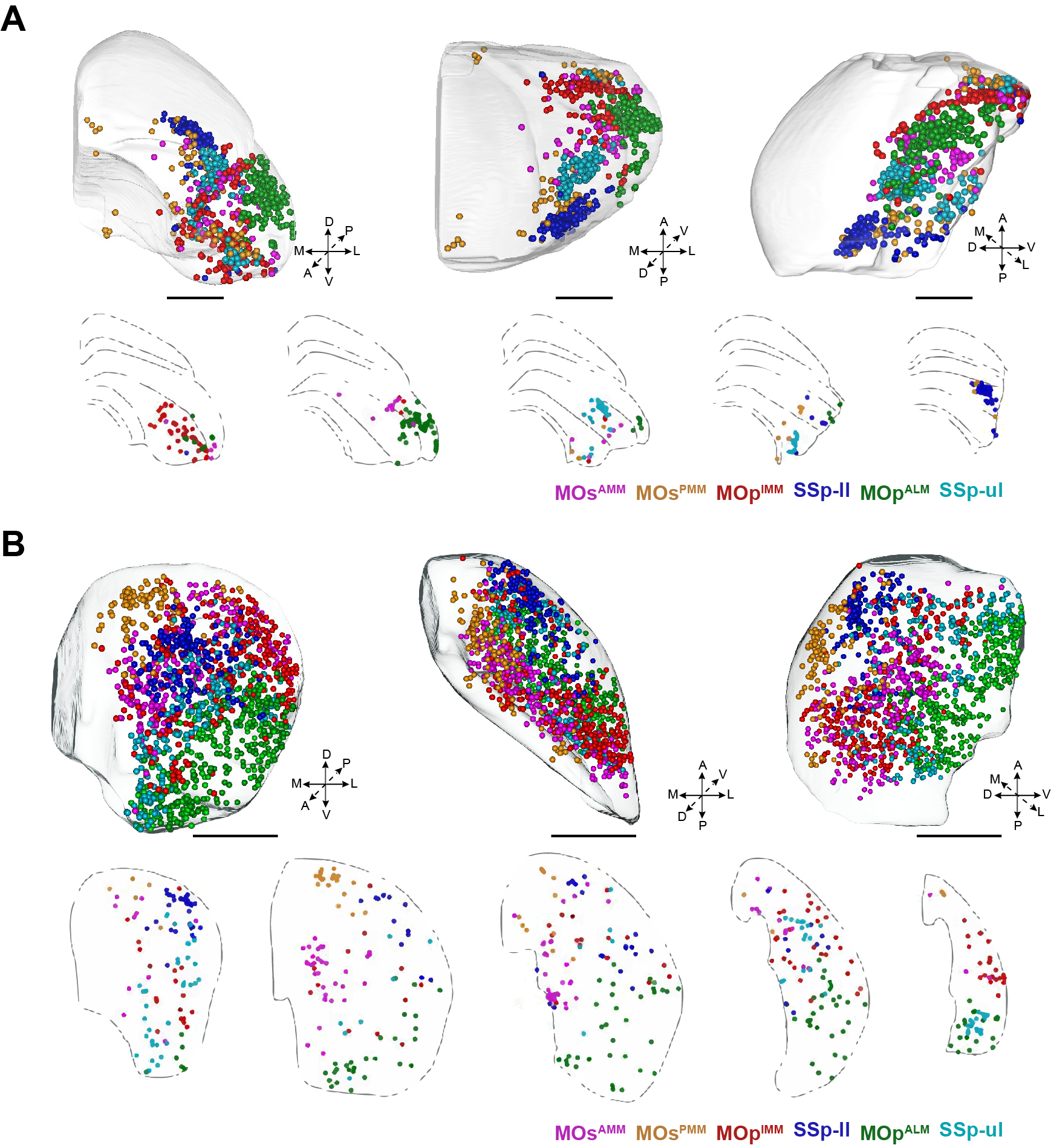

Supplement: Supplementary 1 — Figs. S1 to S13 Tables S1 to S3 Materials and Methods References [57–59] [file research.0470.f1.zip › Sup_Figure8.jpg]

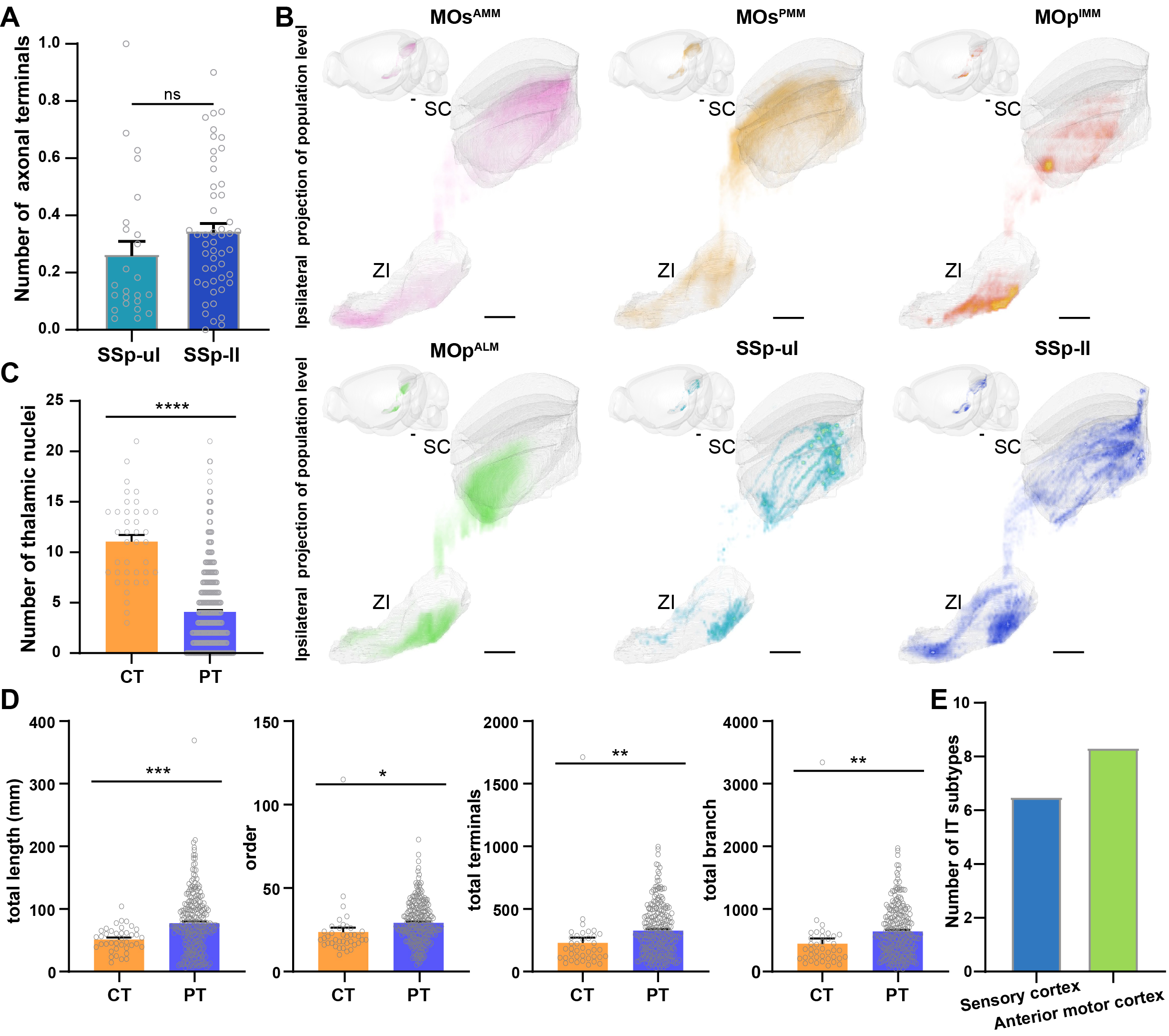

Supplement: Supplementary 1 — Figs. S1 to S13 Tables S1 to S3 Materials and Methods References [57–59] [file research.0470.f1.zip › Sup_Figure9.jpg]
